# Sequencing and Validation of Reference Genes to Analyze Endogenous Gene Expression and Quantify Yellow Dwarf Viruses Using RT-qPCR in Viruliferous *Rhopalosiphum padi*

**DOI:** 10.1371/journal.pone.0097038

**Published:** 2014-05-08

**Authors:** Keke Wu, Wenwen Liu, Thithi Mar, Yan Liu, Yunfeng Wu, Xifeng Wang

**Affiliations:** 1 College of Plant Protection, Northwest A & F University, Yangling, Shaanxi, China; 2 State Key Laboratory for Biology of Plant Diseases and Insect Pests, Institute of Plant Protection, Chinese Academy of Agricultural Sciences, Beijing, China; Department of Primary Industries and Fisheries, Australia

## Abstract

The bird cherry-oat aphid (*Rhopalosiphum padi*), an important pest of cereal crops, not only directly sucks sap from plants, but also transmits a number of plant viruses, collectively the yellow dwarf viruses (YDVs). For quantifying changes in gene expression in vector aphids, reverse transcription-quantitative polymerase chain reaction (RT-qPCR) is a touchstone method, but the selection and validation of housekeeping genes (HKGs) as reference genes to normalize the expression level of endogenous genes of the vector and for exogenous genes of the virus in the aphids is critical to obtaining valid results. Such an assessment has not been done, however, for *R. padi* and YDVs. Here, we tested three algorithms (GeNorm, NormFinder and BestKeeper) to assess the suitability of candidate reference genes (EF-1α, ACT1, GAPDH, 18S rRNA) in 6 combinations of YDV and vector aphid morph. EF-1α and ACT1 together or in combination with GAPDH or with GAPDH and 18S rRNA could confidently be used to normalize virus titre and expression levels of endogenous genes in winged or wingless *R. padi* infected with Barley yellow dwarf virus isolates (BYDV)-PAV and BYDV-GAV. The use of only one reference gene, whether the most stably expressed (EF-1α) or the least stably expressed (18S rRNA), was not adequate for obtaining valid relative expression data from the RT-qPCR. Because of discrepancies among values for changes in relative expression obtained using 3 regions of the same gene, different regions of an endogenous aphid gene, including each terminus and the middle, should be analyzed at the same time with RT-qPCR. Our results highlight the necessity of choosing the best reference genes to obtain valid experimental data and provide several HKGs for relative quantification of virus titre in YDV-viruliferous aphids.

## Introduction

*Rhopalosiphum padi* L. (Hemiptera: Aphididae) is an important pest of wheat, oat, barley, rye and other gramineous crops in the world [1], [2]. It can sometimes directly affect grain yield by sucking nutrients from these plants, but causes the most serious damage when it transmits a group of viruses that are collectively called yellow dwarf viruses (YDVs) to cereal crops and grasses [3], [4]. *R. padi* is considered a competent vector of the YDVs, which comprise Barley yellow dwarf virus (BYDV)-PAV and BYDV-PAS (genus *Luteovirus*) and Cereal yellow dwarf virus (CYDV)-RPV, CYDV-RPS (genus *Polerovirus*) and ungrouped BYDV-GPV [renamed Wheat yellow dwarf virus (WYDV)-GPV] [4], [5], [6], [7], all of which belong to the family *Luteoviridae* [8].

*R. padi* transmits YDVs in a persistently circulative and nonpropagative mode [9], [10] after piercing a YDV-infected plant and ingesting virions while sucking up the phloem sap [11]. Receptor-mediated endocytosis and release must then occur in the midgut and/or the hindgut and in the accessory salivary glands (ASG) [12], [13]. Otherwise, the virions will be excreted along with the feces or be retained in the aphid and exposed to a destructive environment or immune elements of the aphid or ill-defined metabolic processes so that it cannot complete the circulative route in the aphid [14]. The propagation of YDVs in their plant hosts has been investigated and elaborated using the prevailing absolute and relative quantification methods [15], [16], but rarely has research focused on fluctuation in the virus titre and accumulation in the vector aphid during the acquisition access period (AAP), latent period or inoculation access period (IAP), all pivotal issues that affect rates of transmission, duration of transmissibility and epidemiology of the plant virus in the field. With the entry of the virus into the vector aphid, many proteins are incorporated via a transcytosis mechanism associated with virus transmission [17], [18], [19], [20], followed by up- or down-regulation of the expression of numerous genes [21]. Using reverse transcription-quantitative polymerase chain reaction (RT-qPCR), we can rapidly assess the influence of virus entry on molecular functions and biological processes and on the real-time concentration of virus [22], [23].

A relative quantification method for many aspects of molecular research has come into favor to eliminate systemic errors among experimental treatments, developmental stages, RNA extraction efficiency, and RNA integrity [24], [25]. With this method, changes in the expression level of genes of interest (GOIs) can be determined by comparing the expression of the GOIs with that of housekeeping genes (HKGs) [24]. HKGs are constitutive in all cells under both normal and disturbed conditions and expressed constantly in natural situations [26]. If the expression of HKGs is validated as remaining constant under a defined set of conditions, relative quantification of GOIs can be realized with convincing results [24]. The selection of HKGs for normalization of the expression of the GOIs has become routine in studies on human disease diagnosis [27], [28]. With the publication of the complete genomes of different insects, from *Drosophila melanogaster* [29] to *Plutella xylostella* L. [30], these holometabolous insects have been the focus of searches for HKGs to use as reference genes [31], [32], [33]. Estimating the level of expression of HKGs to assay differences in expression in hemimetabolous insects, such as aphids, which transmit many plant viruses, has started recently with *Aphis glycines* [34]. For detecting YDV virions in the vector *R. padi* and determining the relative level of expression of vector genes using relative RT-qPCR, no HKG has been validated as a suitable reference gene to normalize expression. Although ACT1 was used to determine two YDVs in *R. padi*, it was actually from a coordinate of *Bombyx mori*, but the ACT1 gene sequence of *R. padi* was unknown [35], [36].

Selecting appropriate and reliable HKGs as reference genes is required for RT-qPCR experiments. Software tools to estimate and validate reference genes include GeNorm [25], Normfinder [37], and BestKeeper [38]. HKGs such as 18S rRNA, ACT1, EF-1α, GAPDH, UBI, RPSs, RPLs and TUB are commonly used reference genes for standardization when analyzing the expression of insect genes using RT-qPCR [34], [39], [40], [41], [42], [43], [44].

The announcement of the complete genome of the pea aphid, *Acyrthosiphon pisum* (Harris) [45], has opened a new avenue for finding homologous genes in other aphid species. The HKGs of *R. padi* can be chosen and cloned based on sequences from *A. pisum*, then used as reference genes to quantify the relative titre of viruses ingested by *R. padi* and the relative expression values of endogenous genes in the YDV-viruliferous aphid, replacing methods for absolute quantification [46], which may lead to incorrect, misleading conclusions [15].

Previously, actin protein and GAPDH protein of *Myzus persicae* were found to possess interaction with *Beet western yellows virus in vitro* [18]. In a recent study, some ACT genes (ACT1 to ACT4) and one GAPDH gene, together with many other genes from *A. pisum* were considered to be potentially related to the transmission of *Pea enation mosaic virus* and *Soybean dwarf virus* [21]. Here, we selected these two doubtful HKG genes, ACT1 and GAPDH, as well as EF-1α and 18S rRNA which were always used as reference genes for normalization of target genes in other aphids [42], [47], as four candidate reference gene for relative RT-qPCR analysis in *R. padi*. Firstly, these four HKGs from *R. padi* were cloned, and then their nucleic acid sequences were aligned with coordinates from other insect species on NCBI. We evaluated the expression stability of these four candidate reference genes using 3 tools (GeNorm, NormFinder, and BestKeeper) in 6 experimental virus–aphid morph combinations after various durations of aphid feeding on oat plants infected with the respective virus. To further explore the applicability of the optimal reference genes selected by GeNorm, we monitored the relative titre of the three viruses in the two morphs of *R. padi* during AAP and compared virus titre among the feeding durations using a one-way ANOVA. Also, the expression of two endogenous aphid genes, *ago-1a* and *dcr1*, whose encoded proteins are involved in miRNA pathway in aphids and have similar functional domains with the RNAi pathway-related Ago-2 and Dcr2 proteins [48], [49], [50], were normalized using the selected reference genes to detect any change in expression.

## Results and Discussion

### Sequencing of Candidate Reference Genes from *R. padi*

The total RNAs extracted from plants and aphids were all of good integrity (Figure S1) and purity (A_260_/A_280_ values: 1.95 ∼ 2.10; A_260_/A_230_ values > 2.0). All genes were amplified by RT-PCR (Figure S2) with the primers described in Table 1. Nucleic acid sequences of 4 candidate reference genes yielded good matches in a BLAST search of the NCBI database with high similarity to corresponding genes from other aphids and insects: 18S rRNA, 90–100%; EF-1α, 83–94%; ACT1, 82–96%; GAPDH, 78–95%, respectively (Table S1).

**Table 1:** Primers for RT-PCR amplification used in this study.

Gene	Forward (F) and reverse (R) primer (5′-3′)	EST size (bp)	Accession number
18s rRNA	F: CTGGTTGATCCTGCCAGTAGTCATATG	571	KJ612093
	R: TTCCGATTACGGGGCCTCGGATG		
ACT1	F: ATGTGTGACGAAGAAGTAGC	1131	KJ612090
	R: TTAGAAGCACTTTCTGTGC		
EF-1α	F: ATTGATATTGCTTTATGGAAATTCG	837	KJ612092
	R: ACCAGGGTGGTTCAATACAATAAC		
GAPDH	F: ATGTCAAACATTGGTATCAATGGATTTGG	999	KJ612091
	R: TTTAATCCTTAGATTGCATGTACTTGAT		
*ago-1a*-1	F: AATCATTTCCAAATTTCAATGCCTCG	727	KJ612094
	R: TTGTAACATTACAAACTCTATATTTTC		
*ago-1a*-2	F: ATTAATTCTTTGGTTCGCCGAGCTG	906	KJ612095
	R: GTACAATATAATTCGATGAGGCTTATAAC		
*ago-1a*-3	F: ATAAAGTTGGAAGGTGATTACAAAC	278	KJ612096
	R: TACGTTAAGCATTGTAATTCATCTG		
*dcr1*	F: TTATGGACTTCCAGACATTAATATTTTATC	957	KJ612097
	R: TGAGTCATTGCTTGTAATAGATAACTACG		
BYDV-GPV CP	F: ATGAGTACGGTCGCCCTTAGAAATG	606	
	R: CTATTTCGGGTTTTGAAACAGGACC		
BYDV-GPV RTD	F: GTAGACGCGGAACCCGGTCCTAG	1215	
	R: TTAGGAACGGCCACCACCAAATG		
BYDV-PAV CP a	F: ATGAATTCAGTAGGCCGTAGAGGAC	600	
	R: GGTTCCGGTGTTGAGGAGTCTAC		
BYDV-PAV RTD b	F: GCCAAATAGGTAGACTCCTCAACA	1341	
	R1: TCACGAAAATTGTGCCTTGTACTC		
	R2: TCACTGAAACTGCGCCTTGTATTC		
	R3: TCACGAGAATTGAGCCTTGTACGC		
BYDV-GAV CP	F: ATGAATTCAGTAGGCCGTAG	600	
	R: CTATTTGGGAGTCATGTTG		
BYDV-GAV RTD	F: GTAGACTCCTCAACACCAGAG	1377	
	R: TCACCATGTGTCAGCTAAAC		

a With this primer pair, RT-PCR product included less than 100 nt of the 5′-terminus of the RTD gene.

b With this primer pair, RT-PCR product included less than 100 nt of the 3′-terminus of the CP gene.

### Expression Profiles of Candidate Reference Genes

**Figure 1:**
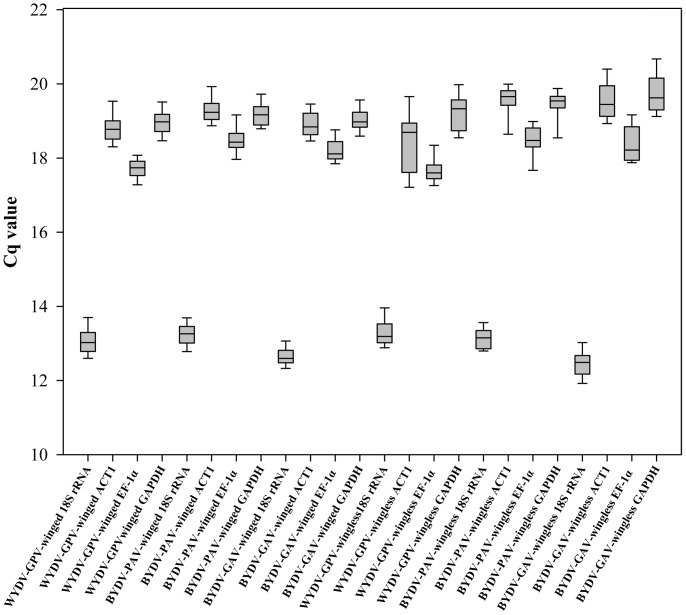
Box and whisker plots of Cq values for the 4 candidate reference genes in 6 experimental groups (one of three viruses in either winged or wingless adults of *Rhopalosiphum padi*). Each box shows the lower 25^th^ and upper 75^th^ percentiles with median Cq values; the whiskers mark the lower 5^th^ and upper 95^th^ percentiles of the Cq values in each data set. Experimental groups from left to right: WYDV-GPV in winged adult, BYDV-PAV in winged adult, BYDV-GAV in winged adult, WYDV-GPV and wingless adult, BYDV-PAV and wingless adult, and BYDV-GAV and wingless adult.

In the two-step RT-qPCR, the amplification efficiencies of the 4 candidate reference genes ranged from 91% to 97% (Table 2; Figure S3). Relative standard curves for determining the amplification efficiencies of these genes are shown in Figure S4, and all Cq values of the dilution series of the cDNA for each gene were less than 30, while those of the no-template controls were either higher than 36 or undetermined. Consequently, the raw Cq value of the individual gene reflected the actual mRNA level of this gene and could be compared directly among the 6 experimental groups. The distribution of the Cq values of the 4 candidate reference genes showed that the expression of 18S rRNA was maintained at extremely high levels with the lowest Cq values (Cq = 12∼14), higher levels with moderate Cq values (Cq 16∼22) were obtained for ACT1, EF-1α and GAPDH, but EF-1α was at the lower edge of this range in all experimental groups (Figure 1). The high expression level of 18S rRNA in *R. padi* was expected, in agreement with the lowest Cq value in other insects [44]. EF-1α, a well-known HKG in various organisms [51] and an evolutionarily conserved nuclear gene in aphids [52], [53], [54], can be used to classify the population of *R. padi* into various subgroups depending on EF-1α diversity in the population [52], [53]. Its expression level was consistent between winged aphids and wingless aphids, lower than that of 18S rRNA but slightly higher than that of ACT1 and GAPDH. The expression levels of ACT1 and GAPDH were similar in *R. padi*, but the mRNA level of ACT1 largely differs from GAPDH in planthoppers [44]. ACT1 and GAPDH encode a cytoskeletal structural protein and a catalytic enzyme, respectively [44]. Thus, it was difficult to interpret here why these two genes with diverse functions actually have similar expression levels in *R. padi*.

**Table 2:** Primers for RT-qPCR amplification used in this study.

Gene	Forward and reverse primer sequence^a^	Amplicon	Efficiency^c^
		size^b^ (bp)	(winged/wingless)
18S rRNA	5′-CGGGAGGAACGCTTTTATTAGA-3′	262	90.506/91.014
	5′-TTGGATGTGGTAGCCGTTTCTC-3′		
ACT1	5′-AACGGAAGCACCTTTGAACC-3′	385	93.665/95.067
	5′-GGAAGAAGCAGCAGTAGCCAT-3′		
EF-1α	5′-TAGACGCTATCCTACCCCCCA-3′	328	95.902/97.157
	5′-GTGAAATCAGCAGCACCCTTG-3′		
GAPDH	5′-ACTACTGTTCACGCTACCACCG-3′	272	94.814/96.216
	5′-GCTGCTTCCTTGACCTTACCTT-3′		
*ago-1a*-1	5′-ACCAGTCTGTTCGTCCATCTCAAT-3′	138	91.891/91.594
	5′-GTTTTCTTTGTTCACCAATGTCCC-3′		
*ago-1a*-2	5′-ACCGAGCATCAGACCTAAAGTATT-3′	215	98.391/99.05
	5′- TTAGAAGTTCCCTGACCATAGAGC-3′		
*ago-1a*-3	5′-TATTCTGTGCCGACAAAAAGG-3′	184	97.263/91.749
	5′-GATTCAAAATGGTTGTCATCCC-3′		
*dcr1*	5′-CTACCACCCTGTTACTTTGTCCC-3′	168	93.875/101.698
	5′-GTCGTCATTTGAGAAGCCCAC-3′		
BYDV-GPV CP	5′-TTCGTTTTCGCAAAGGATTCA-3′	359	94.75/101.875
	5′-GTGTTGGCGTCACCGTTACC-3′		
BYDV-GPV	5′-GACCATCACCCACTCCACCT-3′	369	102.875/96.203
RTD	5′-CCTCGCTTGAATCATCATACG-3′		
BYDV-PAV CP	5′-CGGGGCTGAGGTATTCGTAT-3′	281	93.65/98.105
	5′-AGGACTTTGAGGCGGATTTG-3′		
BYDV-PAV	5′-CATTGCCTACTCCAACTCATCG-3′	288	100.303/92.586
RTD	5′-ATTCTTTTGTTCGTGTACCCTCC-3′		
BYDV-GAV CP	5′-CAGGCAGGACTGAGGTATTCGTA-3′	278	96.752/100.563
	5′-GGTTGCTGATTTTGAGATGGTGA-3′		
BYDV-GAV	5′-GTGCTGACCCCAAGTTTTACCTC-3′	191	98.585/100.127
RTD	5′-GTTTTCTGTTTTCCTAACCGTGCT-3′		

a Designed by an automated search in Primer Premier 5 software. Parameters for each primer pair: G+C content = 40–60%, annealing temperature between 58° and 62°C, and no false priming.

b Amplicon size and specificity were checked using melt curve and 2% agarose gel electrophoresis (Figure S3).

c Efficiency % = 10^(−1/slope)^ −1. *R* ^2^ values of all relative standard curves were more than 0.989.

The influence of virus and aphid morph on the Cq values of the 4 candidate reference genes was statistically analyzed with a two-way ANOVA (Table 3). When comparing Cq values for each gene (18S rRNA, ACT1 and EF-1α) among the different YDV-viruliferous aphids, Cq differed significantly (all *p*<0.001); values for GAPDH differed less among the three viruses (0.01<*p*<0.05). For the aphid morph factor, wing morph had no significant effect on the expression of 18S rRNA (*p* = 0.285) or EF-1α (*p* = 0.874), but did contribute to the significant difference in ACT1 (*p* = 0.04) and in GAPDH (*p*<0.001). For the interaction of YDV and wing morph, Cq values of 18S rRNA and EF-1α revealed no significant difference (both *p*>0.05), as opposed to the Cq values for ACT1 (*p*<0.001) and GAPDH (*p* = 0.02), probably due to the significant differences for both virus and wing morph.

**Table 3:** Influence of YDV entry and wing morph on raw Cq value for individual genes.

Gene	Virus	Wing morph	Interaction
18S rRNA	0.000***	0.285^ns^	0.276^ns^
ACT1	0.000***	0.040*	0.000***
EF-1α	0.000***	0.874^ns^	0.504^ns^
GAPDH	0.010*	0.000***	0.020*

Two-way ANOVA analysis: ns, nonsignificant, *p*>0.05; * *p*<0.05, ** *p*<0.01; *** *p*<0.001.

Since the virus was considered the main effect and we had more than 2 viruses, we used Tukey’s honestly significant difference (HSD) post-hoc test to compare the influence of (1) BYDV-GAV vs. WYDV-GPV, (2) BYDV-GAV vs. BYDV-PAV, and (3) WYDV-GPV vs. BYDV-PAV on each candidate reference gene (Table 4). For the influence of infection by BYDV-GAV vs. WYDV-GPV on the expression of 18S rRNA, ACT1 or EF-1α of *R. padi*, the expression levels of these candidate reference genes differed significantly (all *p*<0.001), and less so for GAPDH (*p* = 0.009). Differences in 18S rRNA expression for BYDV-GAV vs. BYDV-PAV was highly significant (*p*<0.001), but not significant for the other 3 reference genes (ACT1, *p* = 0.310; EF-1α, *p* = 0.139; GAPDH, *p* = 0.590). WYDV-GPV and BYDV-PAV differed significantly in expression of ACT1 and of EF-1α (both *p*<0.001), but consistently lacked differences for 18S rRNA (*p* = 0.879) and for GAPDH (*p* = 0.109).

**Table 4:** Paired comparisons for reference genes between the WYDV-GPV (GPV), BYDV-PAV (PAV) and BYDV-GAV (GAV)-viruliferous aphids (*Rhopalosiphum padi*).

	GAV		GPV		PAV	
Gene	GPV	PAV	GAV	PAV	GAV	GPV
18S rRNA	0.000***	0.000***	0.000***	0.879 ^ns^	0.000***	0.879 ^ns^
ACT1	0.000***	0.310 ^ns^	0.000***	0.000**	0.310 ^ns^	0.000***
EF-1α	0.000***	0.139 ^ns^	0.000***	0.000**	0.139 ^ns^	0.000***
GAPDH	0.009**	0.590 ^ns^	0.009**	0.109 ^ns^	0.590 ^ns^	0.109^ ns^

Tukey’s HSD post hoc tests: ns, nonsignificant, *p*>0.05; * *p*<0.05; ** *p*<0.01; *** *p*<0.001.

In summary, the Cq values for expression of 18S rRNA in WYDV-GPV- and BYDV-PAV-viruliferous *R. padi* fell into one group (subset 2), the BYDV-GAV-viruliferous *R. padi* into another (subset 1). As for the Cq values of ACT1 and EF-1α, the WYDV-GPV-viruliferous *R. padi* was in one group (subset 1), the others in a second (subset 2). The Cq values for the GAPDH in BYDV-PAV- and BYDV-GAV-viruliferous *R. padi* are logically within one group, rather than with WYDV-GPV-viruliferous *R. padi*, because of the lack of significant differences between the infection with BYDV-PAV and BYDV-GAV (*p* = 0.590), with *p*-values lower than that for the comparison between WYDV-GPV and BYDV-PAV (*p* = 0.109). The lack of unanimous grouping of Cq values for these 4 candidate reference genes might be attributed to the differing transmission properties of the YDVs by *R. padi* because successful transmission depends on the compatibility between the virus and vector insects [9].

### Expression Stability Analysis of Reference Genes in YDV-viruliferous *R. padi*

For analyzing the expression stability of the 4 candidate reference genes, a stability value for each individual gene was calculated using three Excel-based tools (GeNorm [25] and NormFinder [37] and BestKeeper [38]). The amplification efficiencies of 4 candidate genes in winged *R. padi* differed somewhat from that of the wingless (Table 2). Therefore, the stability ranking fell into two sets.

GeNorm only recognizes linear scale quantities, obtained by transforming raw Cq values using the delta-Cq method (described in the methods) or a standard curve; an expression stability value *M* for all genes contained in the InputData file is automatic calculated by an algorithm [25] and displayed. The smallest *M* value indicates the most stable gene expression; the largest *M* value indicates the most unstable expression. Importantly, expression of a gene with *M* >1.5 is considered to be inconsistent [25]. After the Cq values for the RT-qPCR of all 4 candidate reference genes under the 6 experimental conditions were transformed into a valid data set (see formula in the methods), the *M* values were calculated by a GeNorm applet. As shown in Tables 5 and 6, 4 virus–aphid combinations (WYDV-GPV-winged, BYDV-GAV-winged, BYDV-PAV-wingless and BYDV-GAV-wingless) had the same order of expression stability from the most stable to the least stable gene: EF-1α, ACT1, GAPDH and 18S rRNA. For the BYDV-PAV-winged combination, ACT1 was ranked as the best reference gene (lowest *M*), followed by EF-1α, GAPDH and 18S rRNA. However, ACT1 was the worst in the case of WYDV-GPV-wingless, and the stability sequence of the other 3 genes was the same for BYDV-PAV-winged. In brief, EF-1α (5 first places and 1 second place) seemed to be the best reference gene to normalize the GOIs in all YDV-viruliferous types of *R. padi*.

**Table 5:** Stability rankings according to three software tools for individual endogenous reference genes in winged adults of *Rhopalosiphum padi* carrying one of three YDVs.

Virus and reference gene	GeNorm a	NormFinder^b^	BestKeeper^c^
	*M*	stability value	Cq set SD
GPV			
EF-1α	0.396	0.080	0.29
ACT1	0.406	0.085	0.34
GAPDH	0.421	0.110	0.30
18S rRNA	0.629	0.192	0.45
PAV			
ACT1	0.270	0.054	0.31
EF-1α	0.274	0.049	0.31
GAPDH	0.309	0.094	0.28
18S rRNA	0.444	0.165	0.26
GAV			
EF-1α	0.211	0.051	0.29
ACT1	0.226	0.074	0.31
GAPDH	0.259	0.076	0.27
18S rRNA	0.338	0.151	0.21

a According to GeNorm, the smallest *M* value indicates the most stable gene expression, the largest *M* value the most unstable; expression of a gene with *M* >1.5 is considered inconsistent.

b Like GeNorm, the most stable gene has the lowest stability value, and vice versa.

c Genes with SD <1 can be ranked according to their Cq SD values. The gene most stably expressed has the lowest SD value; the highest SD value indicates the gene with the most unstable expression.

**Table 6:** Stability rankings according to three software tools for individual endogenous reference genes in wingless adults of *Rhopalosiphum padi* carrying one of three YDVs.

Virus and reference gene	GeNorm^a^	NormFinder^b^	BestKeeper^c^
	*M*	stability value	Cq set SD
GPV			
** **EF-1α	0.508	0.033	0.26
GAPDH	0.570	0.115	0.46
18S rRNA	0.648	0.202	0.29
ACT1	0.799	0.231	0.68
PAV			
EF-1α	0.277	0.017	0.36
ACT1	0.285	0.028	0.38
GAPDH	0.296	0.060	0.34
18S rRNA	0.535	0.213	0.24
GAV			
EF-1α	0.293	0.053	0.42
ACT1	0.294	0.054	0.49
GAPDH	0.317	0.078	0.48
18S rRNA	0.527	0.212	0.29

a According to GeNorm, the smallest *M* value indicates the most stable gene expression, the largest *M* value the most unstable; expression of a gene with *M* >1.5 is considered inconsistent.

b Like GeNorm, the most stable gene has the lowest stability value, and vice versa.

c Genes with SD <1 can be ranked according to their Cq SD values. The gene most stably expressed has the lowest SD value; the highest SD value indicates the gene with the most unstable expression.

A different algorithm, NormFinder, only analyzes linear scale data [37]. The stability values of the reference genes can be calculated for one experimental group or for multiple groups in one data set. The most stable gene has the lowest stability value, and vice versa [37]. When the transformed linear data (the same *Q* values of the 4 candidate reference gene analyzed by GeNorm) was analyzed with NormFinder, sorting for expression stability of the candidate reference genes (Tables 5 and 6) for the WYDV-GPV-winged, BYDV-PAV-winged, BYDV-GAV-winged and BYDV-PAV-wingless groups was highly uniform: EF-1α was the most stable gene, but 18S rRNA was the most unstable. EF-1α was also the first choice of reference genes for the WYDV-GPV-wingless group, but sorted as the third most stable gene for the BYDV-GAV-wingless.

Finally, the raw Cq values of candidate reference genes in each group were assessed directly by BestKeeper; when the SD of the Cq is higher than 1, expression for that is considered as inconsistent, and only genes with a SD less than 1 can be ordered using their Cq SD values [38]. The gene most stably expressed thus has the lowest SD value; the gene most unstably expressed has the highest SD value. In this study, Cq SD values from the qPCRs of the 4 candidate reference genes in all groups were less than 1 (Tables 5 and 6) and can be compared with each other as individual groups. Unlike the results from GeNorm and NormFinder, 18S rRNA was evaluated as the first and second most stable gene in the following 5 groups: BYDV-PAV-winged, BYDV-GAV-winged, BYDV-PAV-wingless, BYDV-GAV-wingless and WYDV-GPV-wingless, but as the least stably expressed gene in the GPV-winged group. EF-1α, assessed as a nearly ideal reference gene by GeNorm and NormFinder tools, was ranked as the optimal reference gene only in the WYDV-GPV-winged and -wingless groups. For the other 4 groups, the performance of EF-1α was mediocre.

As discussed already, GeNorm and NormFinder yielded very similar results with respect to sorting expression stability in each group and in identifying the best reference gene among 4 candidate reference genes. We inferred that the two programs analyzed the same linear scale data set, which was transformed from raw Cq values and corrected by amplification efficiency of the respective gene (see formula in the methods). The results from the BestKeeper tool were nearly opposite to those obtained with GeNorm and NormFinder. Perhaps the number and variety of candidate reference genes that we used here are not very diversified.

### Selection of Reference Genes for Normalization

**Figure 2:**
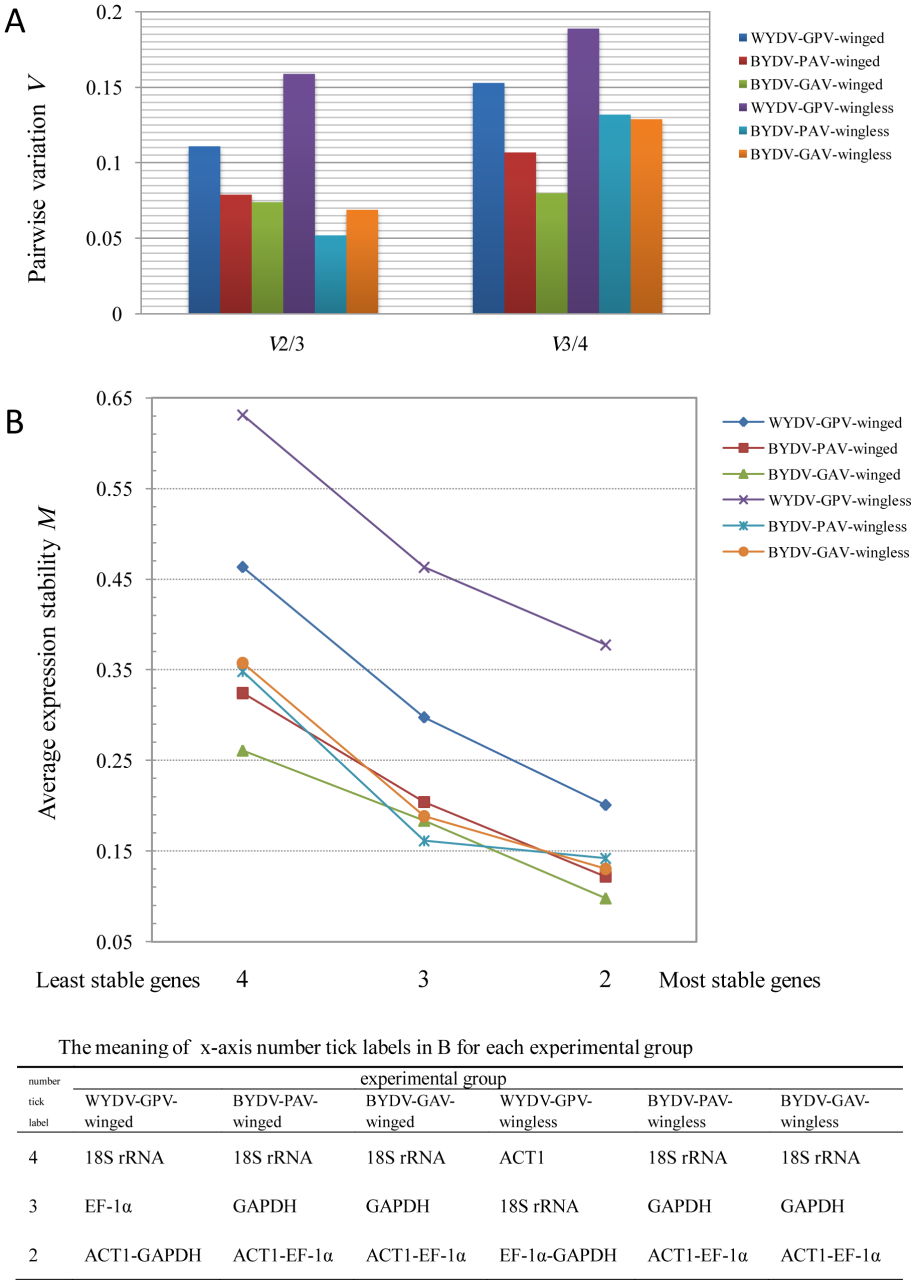
Determination of optimal reference genes for normalization by GeNorm. The optimal number of reference genes was determined by pairwise variation *V* (A) between two sequential normalization factors containing an increasing number of reference genes (*n* and *n* +1 reference genes). The pairwise variation begins with the first two and three (*V*2/3) most stable candidate reference genes (B), terminating with the addition of the least stable one, here *V*3/4. The cut-off *V* value is set at 0.15 by the program GeNorm, below which an additional reference gene does not need to be included for calculating the normalization factor.

There were 3 cases for calculating the best number of reference genes in our 6 experimental groups: (1) *V*2/3<0.15, *V*3/4<0.15; (2) *V*2/3<0.15, *V*3/4>0.15; and (3) *V*2/3>0.15, *V*3/4>0.15 (Figure 2A). The first case meant the best number of reference genes could be 2, 3 or 4; simply, the 2 most stable genes, ACT1 and EF-1α, (Figure 2B) were the ideal combination of reference genes for the normalization analysis of target genes in the respective groups, BYDV-PAV-winged, BYDV-GAV-winged, BYDV-PAV-wingless, and BYDV-GAV-wingless. The second case demonstrated that the optimal choice of reference genes was ACT1 and GAPDH for the WYDV-GPV-winged group (Figure 2B), while adding another one or two candidate reference genes increased the risk of misinterpreting the expression profile of the GOIs. The last case indicated that using just these 4 candidate reference genes would not form a dependable reference gene group for the relative quantification of GOIs in WYDV-GPV-viruliferous wingless *R. padi*.

### Relative Quantification of Endogenous Genes

Since no up- or down-regulation of these candidate internal control genes was observed in the 6 combinations (SD <1 from BestKeeper and stability value *M* <1.5 from GeNorm), and the optimal number of reference genes had been selected by GeNorm, these genes can be used in the expression profile analysis of endogenous and low-abundance genes. Because the expression levels of *ago-1a* and *dcr1a* (*dcr1*) gene varied significantly among four reproductive morphs of *A.* pisum when measured with semi-quantitative RT-PCR [49], miRNA machinery and alternative transcription are thought to play a key role in morph transformation in the life cycle of *A. pisum*.

**Figure 3:**
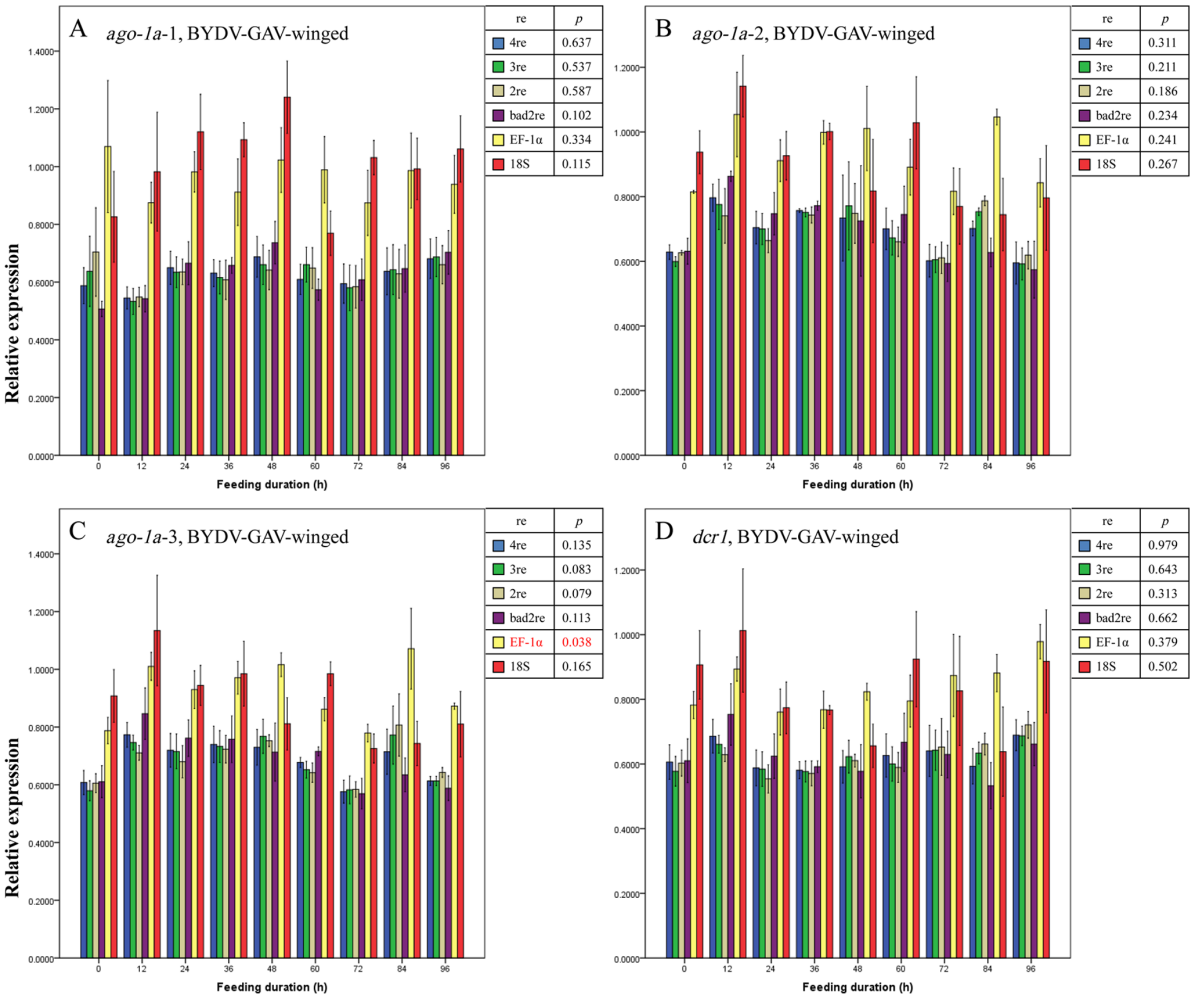
Mean relative expression values (± SE, *n* = 3) for endogenous aphid genes *ago-1a*-1 (A), *ago-1a*-2 (B), *ago-1a*-3 (C) and *dcr1* (D) in BYDV-GAV-viruliferous winged adults of *Rhopalosiphum padi* after different virus-feeding durations. A–D, Expression values for each gene or each region of one gene at each duration were normalized with the reference gene(s) selected by GeNorm and then compared with a one-way ANOVA (*p*) among these durations with each normalization condition. 2re = 2 best reference genes; 3re = 3 best reference genes; 4re = all 4 reference genes; bad2re = 2 least stable reference genes; EF-1α = EF-1α as the reference gene; 18S = 18S rRNA as the reference gene.

**Figure 4:**
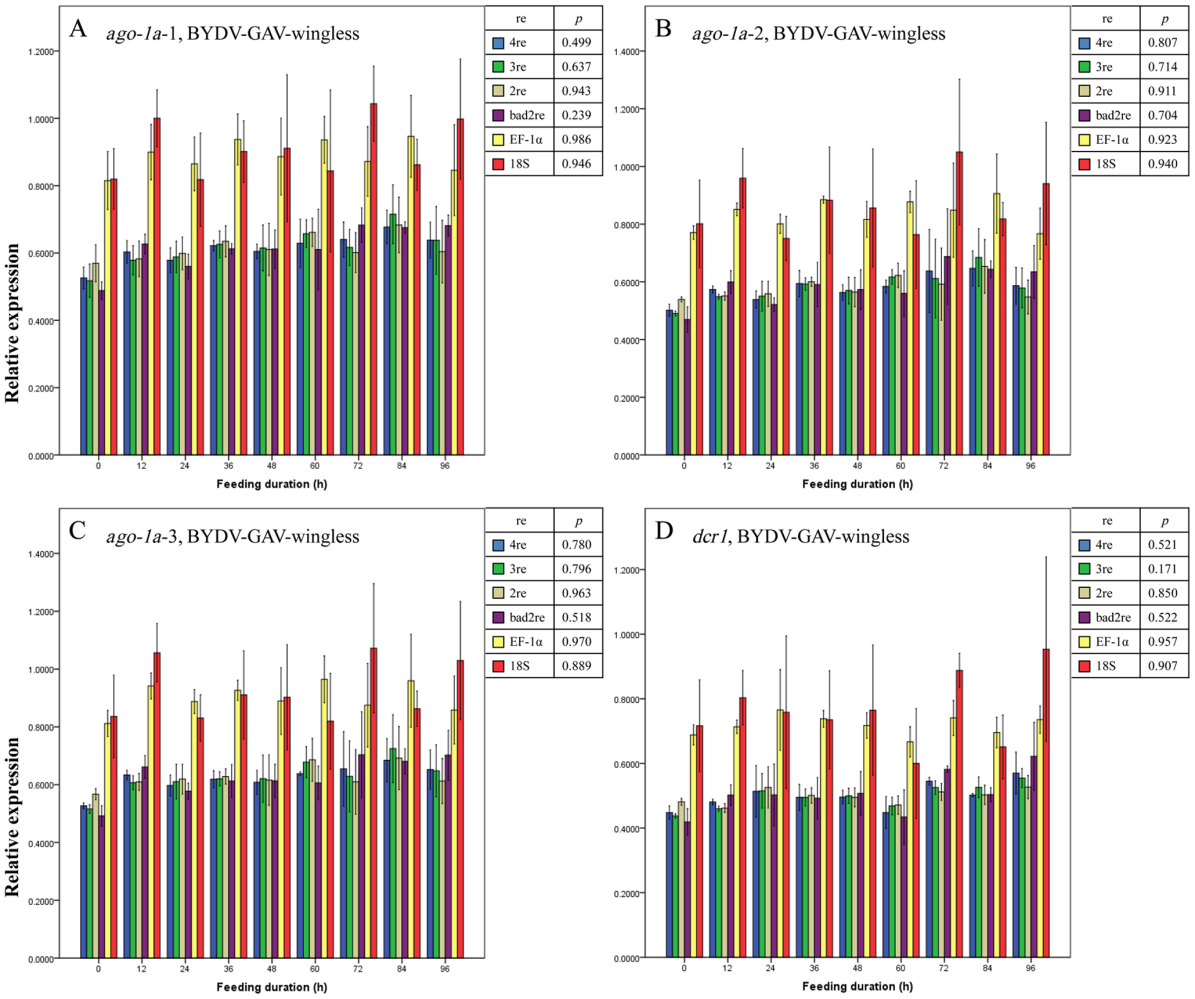
Mean relative expression values (± SE, *n* = 3) for endogenous aphid genes *ago-1a*-1 (A), *ago-1a*-2 (B), *ago-1a*-3 (C) and *dcr1* (D) in BYDV-GAV-viruliferous wingless adults of *Rhopalosiphum padi* after different virus-feeding durations. A–D, Expression values for each gene or each region of one gene at each duration were normalized with the reference gene(s) selected by GeNorm and then compared with a one-way ANOVA (*p*) among these durations with each normalization condition. 2re = 2 best reference genes; 3re = 3 best reference genes; 4re = all 4 reference genes; bad2re = 2 least stable reference genes; EF-1α = EF-1α as the reference gene; 18S = 18S rRNA as the reference gene.

Regardless of the normalization method used (described in Materials and Methods), no significant differences were observed in the expression level for the 3 regions of the *ago-1a* and *dcr1* genes before and after the entry of BYDV-GAV into winged (Figure 3 A–D) or wingless (Figure 4 A–D) adults of *R. padi* (all *p*>0.05, except for when EF-1α was used as the reference gene to normalize the expression change of *ago-1a*-3 region [*p* = 0.038] [Figure 3C]). We thus concluded that the expression of the *ago-1a* and *dcr1* genes did not respond to the entry of BYDV-GAV into *R. padi* and remained unchanged. This finding conformed to the facts that BYDV-GAV could scarcely be attached by receptor proteins of *R. padi*, and has rare chance to access to hindgut apical plasmalemma [12].

**Figure 5:**
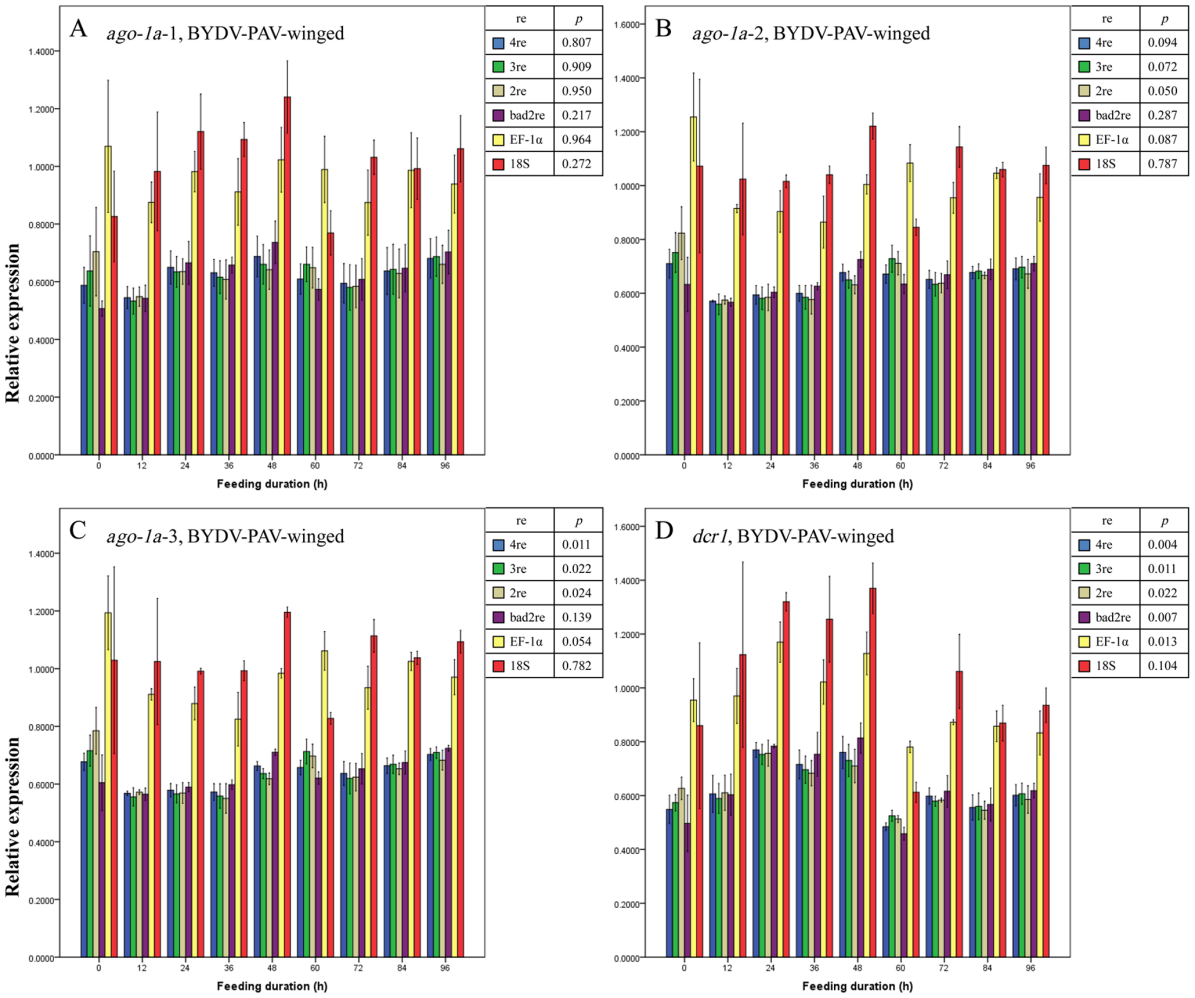
Mean relative expression values (± SE, *n* = 3) for endogenous aphid genes *ago-1a*-1 (A), *ago-1a*-2 (B), *ago-1a*-3 (C) and *dcr1* (D) in BYDV-PAV-viruliferous winged adults of *Rhopalosiphum padi* after different virus-feeding durations. A–D, Expression values for each gene or each region of one gene at each duration were normalized with the reference gene(s) selected by GeNorm and then compared with a one-way ANOVA (*p*) among these durations with each normalization condition. 2re = 2 best reference genes; 3re = 3 best reference genes; 4re = all 4 reference genes; bad2re = 2 least stable reference genes; EF-1α = EF-1α as the reference gene; 18S = 18S rRNA as the reference gene.

**Figure 6:**
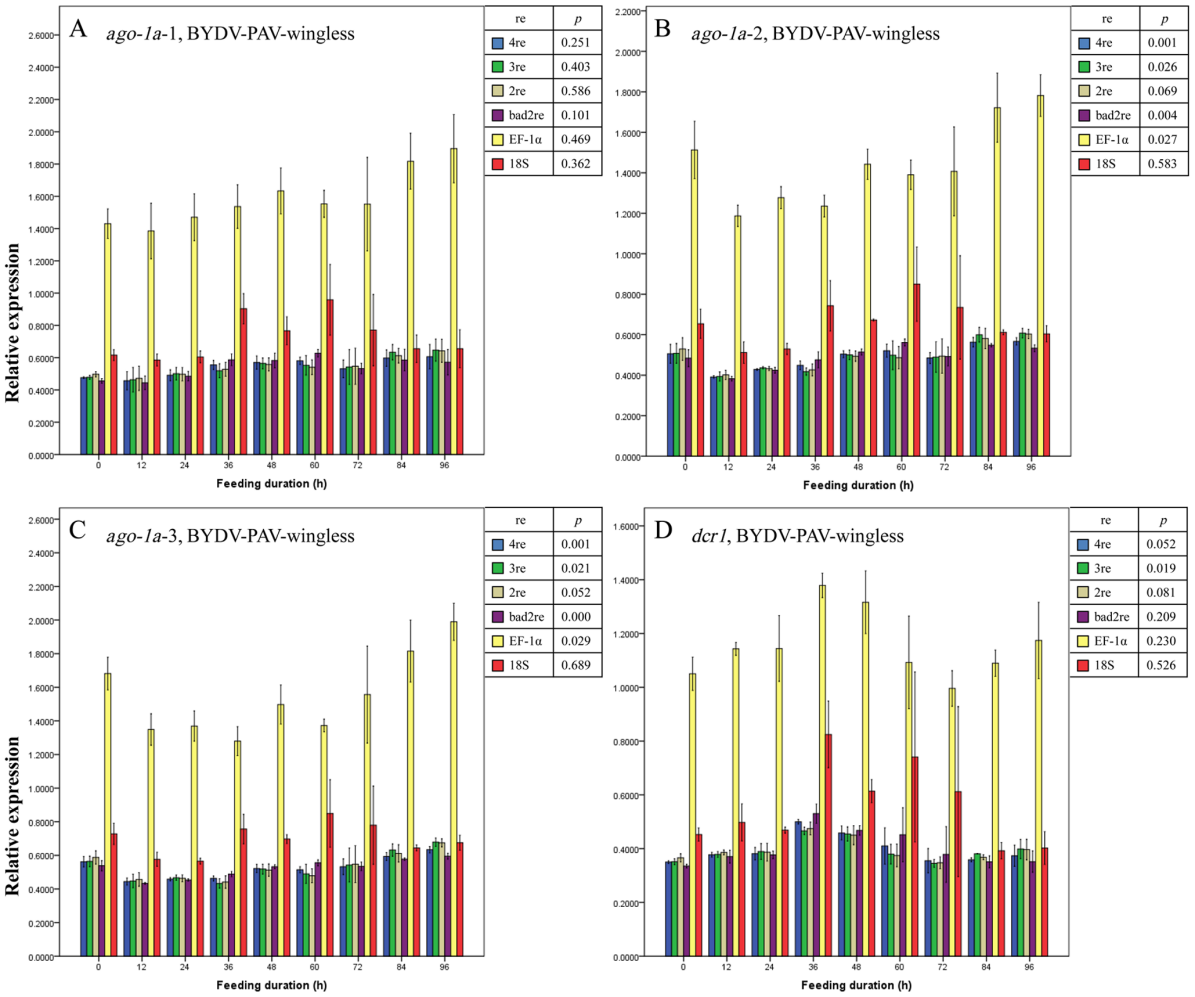
Mean relative expression values (± SE, n = 3) for endogenous aphid genes *ago-1a*-1 (A), *ago-1a*-2 (B), *ago-1a*-3 (C) and *dcr1* (D) in BYDV-PAV-viruliferous wingless adults of *Rhopalosiphum padi* after different virus-feeding durations. A–D, Expression values for each gene or each region of one gene at each duration were normalized with the reference gene(s) selected by GeNorm and then compared with a one-way ANOVA (*p*) among these durations with each normalization condition. 2re = 2 best reference genes; 3re = 3 best reference genes; 4re = all 4 reference genes; bad2re = 2 least stable reference genes; EF-1α = EF-1α as the reference gene; 18S = 18S rRNA as the reference gene.

The entry of BYDV-PAV did not seem to affect the expression profile of *ago-1a* in either of the wing morphs of *R. padi* on the basis of the results in Figures 5A and 6A, for relative expression values of *ago-1a*-1 among these feeding durations approached the same (all *p*>0.05 under all normalizations). However, for expression levels of *ago-1a*-2 and *ago-1a*-3 (the middle region and 3′-terminus of *ago-1a* gene [49]) using all the same normalization methods, we drew different conclusions: after being normalized with a single reference gene (EF-1α or 18S rRNA) or with bad reference genes, the expression of *ago-1a*-2 and -3 in BYDV-PAV-viruliferous winged *R. padi* showed no significant changes from 0 h to 96 h feeding duration (purple, yellow and red bars in Figure 5B and C, all *p*>0.05); thus, *ago-1a*-2 and -3 appeared to be unaffected by BYDV-PAV acquisition; when the best 2 or 3 reference genes or all 4 reference genes were used to normalize the expression of these two regions in winged *R. padi*, a difference in the expression of *ago-1a*-2 among the different feeding durations emerged slightly above the significance level (blue, green, and brown bars in Figure 5B, 0.1>*p*≥0.05), but the expression values for *ago-1a*-3 already declined significantly before (0 h) and immediately after entry of BYDV-PAV (blue, green, and brown bars in Figure 5C, *p*<0.05) in spite of recover to initial level afterwards. In wingless *R. padi*, the changes in expression for *ago-1a*-3 higher than that for *ago-1a*-2 (Figure 6B and C, both *p*<0.1, the *p* value for *ago-1a*-3 was lower than for *ago-1a*-2) was observed when the best 2 or 3 reference genes or all 4 reference genes were used as normalization methods, with a similar trend in the expression change for *ago-1a-*2 and *ago-1a-*3 in the winged morph. On the basis of these findings, we concluded that although no change in expression was detected for *ago-1a*-1 in *R. padi*, the expression of the full-length *ago-1a* was disturbed by the entry of BYDV-PAV particles as seen by the obvious expression change in *ago-1a*-2 and *ago-1a*-3. For the expression change in *dcr1* in BYDV-PAV-viruliferous winged and wingless *R. padi* (Figures 5D and 6D), the significance level (both *p*<0.1) was near that of *ago-1a*-2 and -3 (all *p*<0.1) with the normalization factors calculated by the best 2, or 3 reference genes or all 4 reference genes. The detected region for the *dcr1* gene is also in the middle region and the 3′-terminus of the full-length *dcr1* gene [49], so we speculated that the entry of BYDV-PAV also interfered with the expression of *dcr1*. The unexpected discrepancies among values for changes in relative expression obtained using 3 regions of the same gene in BYDV-PAV-viruliferous *R. padi* suggested that mechanisms on the splicing and reconstruction of these three regions in *ago-1a* were differences in aphids, probably because the pre-mRNA of region 2 and region 3 were recruited and then translate into the PAZ and PIWI domains of antivirus defense-associated AGO2 protein in aphids.

**Figure 7:**
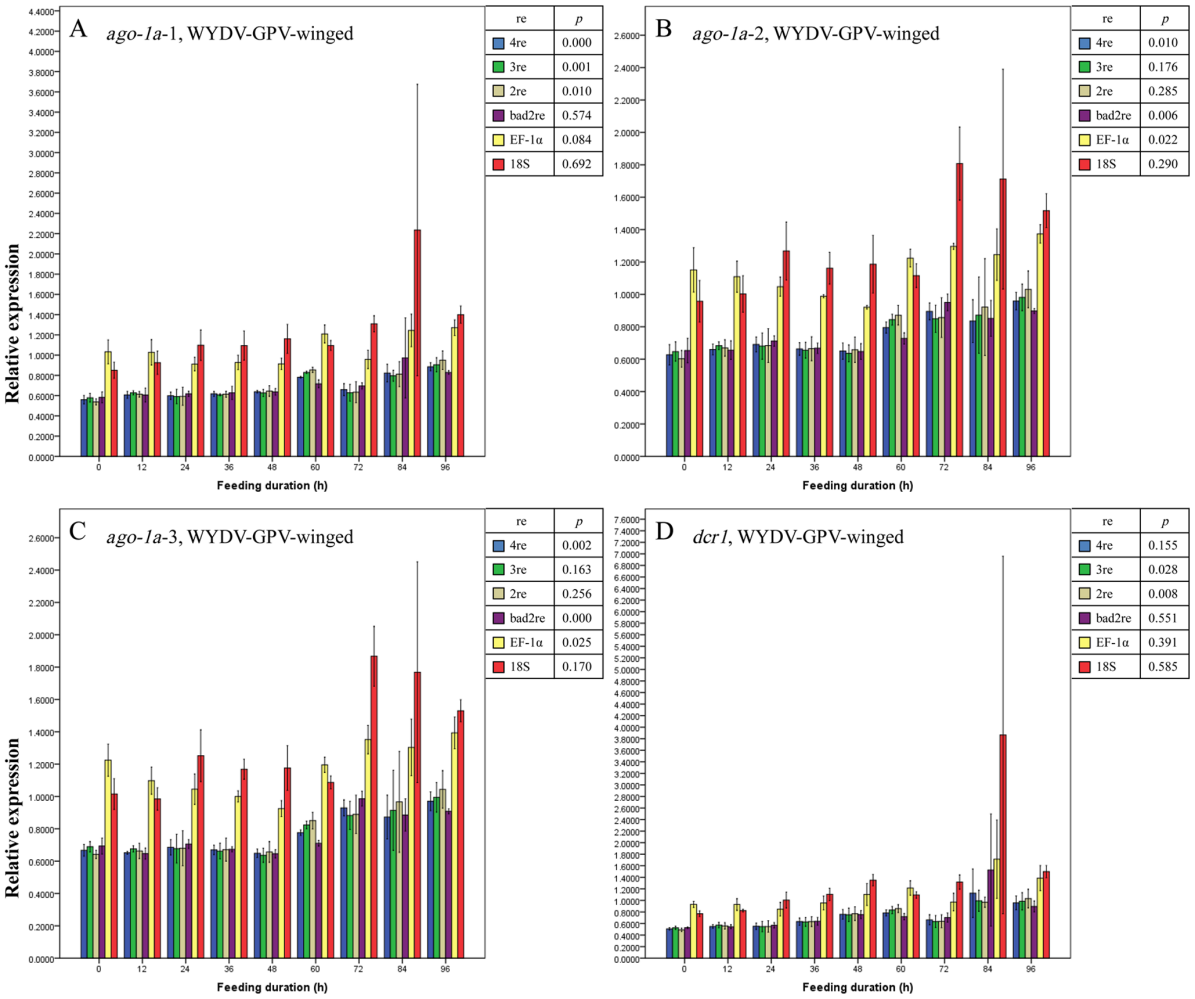
Mean relative expression values (± SE, n = 3) for endogenous aphid genes *ago-1a*-1 (A), *ago-1a*-2 (B), *ago-1a*-3 (C) and *dcr1* (D) in BYDV-GPV-viruliferous winged adults of *Rhopalosiphum padi* after different virus-feeding durations. A–D, Expression values for each gene or each region of one gene at each duration were normalized with the reference gene(s) selected by GeNorm and then compared with a one-way ANOVA (*p*) among these durations with each normalization condition. 2re = 2 best reference genes; 3re = 3 best reference genes; 4re = all 4 reference genes; bad2re = 2 least stable reference genes; EF-1α = EF-1α as the reference gene; 18S = 18S rRNA as the reference gene.

**Figure 8:**
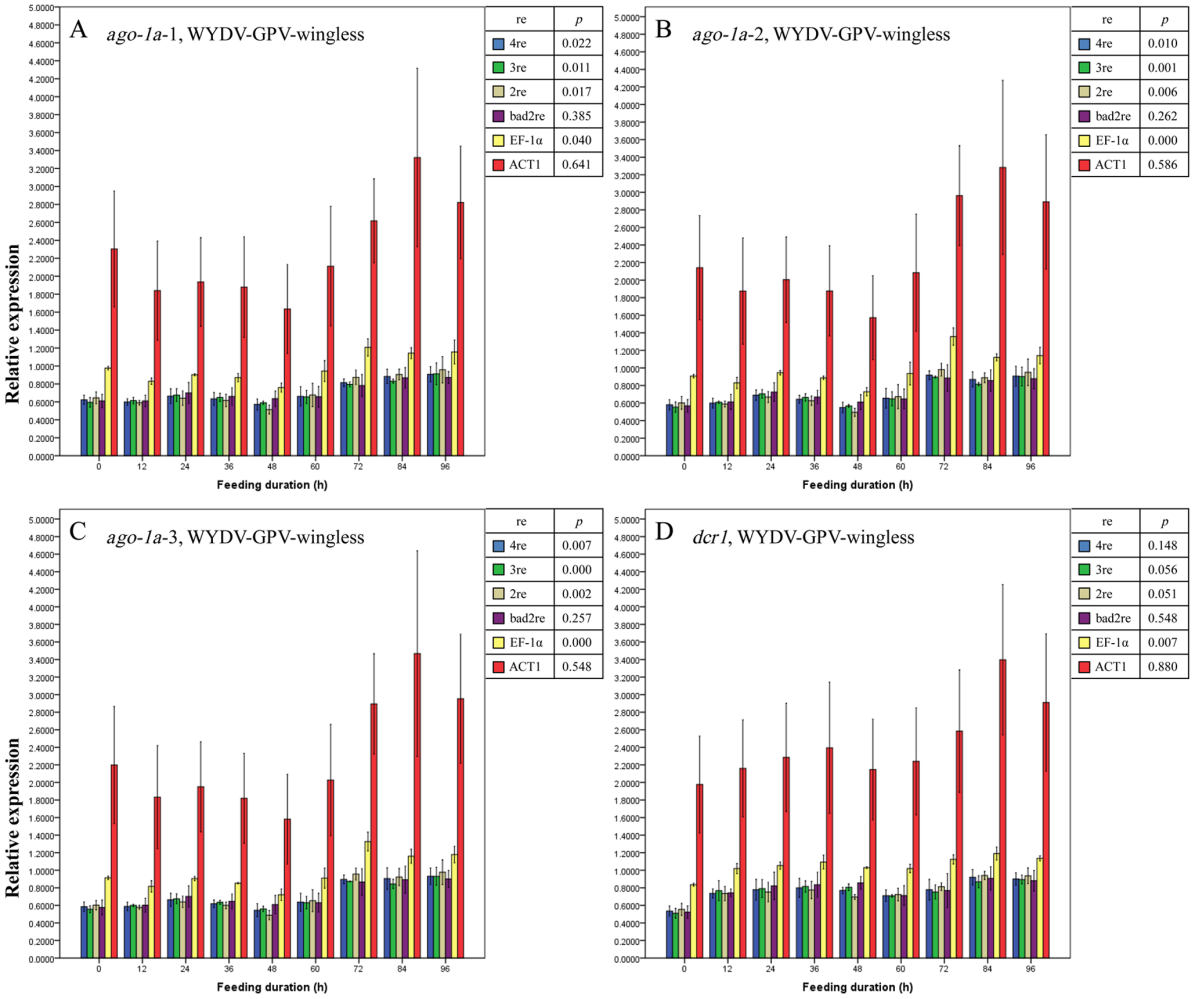
Mean relative expression values (± SE, n = 3) for endogenous aphid genes *ago-1a*-1 (A), *ago-1a*-2 (B), *ago-1a*-3 (C) and *dcr1* (D) in BYDV-GPV-viruliferous wingless adults of *Rhopalosiphum padi* after different virus-feeding durations. A–D, Expression values for each gene or each region of one gene at each duration were normalized with the reference gene(s) selected by GeNorm and then compared with a one-way ANOVA (*p*) among these durations with each normalization condition. 2re = 2 best reference genes; 3re = 3 best reference genes; 4re = all 4 reference genes; bad2re = 2 least stable reference genes; EF-1α = EF-1α as the reference gene; 18S = 18S rRNA as the reference gene.

For WYDV-GPV-viruliferous *R. padi*, we could not give an explicit inference that the relative expression in the 3 regions of *ago-1a* and *dcr1* gene changed or unchanged with the virus acquisition, since these normalization methods provided no concurrent and regular results (Figures 7 and 8). We inferred that the complex interactions between WYDV-GPV, oat plant and vector *R. padi* led to the unsuitability of the selected reference genes (Figure 2A, *V*2/3 and *V*3/4 near or above the cut-off value of 0.15).

The miRNA pathway has close relationship with RNAi pathway in eukaryotes, and the two pathways possess extremely parallel mechanism of RNA cleavage in cell cytoplasm [55]. The machinery proteins of miRNA pathway, Ago-1and Dcr1, have high similarity with those of RNAi pathway, Ago-2 and Dcr2 [49], [56]. Since *R. padi* is efficient vector of BYDV-PAV and WYDV-GPV, these RNA pathways may be triggered against virus invasion [56].

### Fluctuation and Comparison of Virus Titre in *R. padi*

Fluctuation in the virus titre in *R. padi* was monitored by two methods. The first method used the SD values of the raw Cq values (Cq SD) for the CP gene and RTP gene (the Cq values of the no-template controls and 0-h-feeding duration sample of these two genes were either higher than 36 or undetermined and thus not analyzed here) as calculated by BestKeeper. In WYDV-GPV and BYDV-PAV, viruliferous winged and wingless *R. padi*, the Cq SD values of the CP gene and RTD gene were all higher than 1, which was affirmed to be inconstant and statistically significantly different among feeding durations; but the Cq SD values of the CP gene and RTD gene of BYDV-GAV in winged and wingless *R. padi* were lower than 1, with no variance in virus titre during the 4-day AAP (Table S2 and S3). These results agreed with the transmission specificity of these three YDVs by *R. padi*, and the difference in Cq at the initial virus-acquisition duration (maximal Cq value) implied the presence of a sluggish receptor-mediated recognition process at the midgut and/or hindgut [9]. One problem with this method is that the trend for increased virus load is inversely related to the duration of virus-feeding.

**Figure 9:**
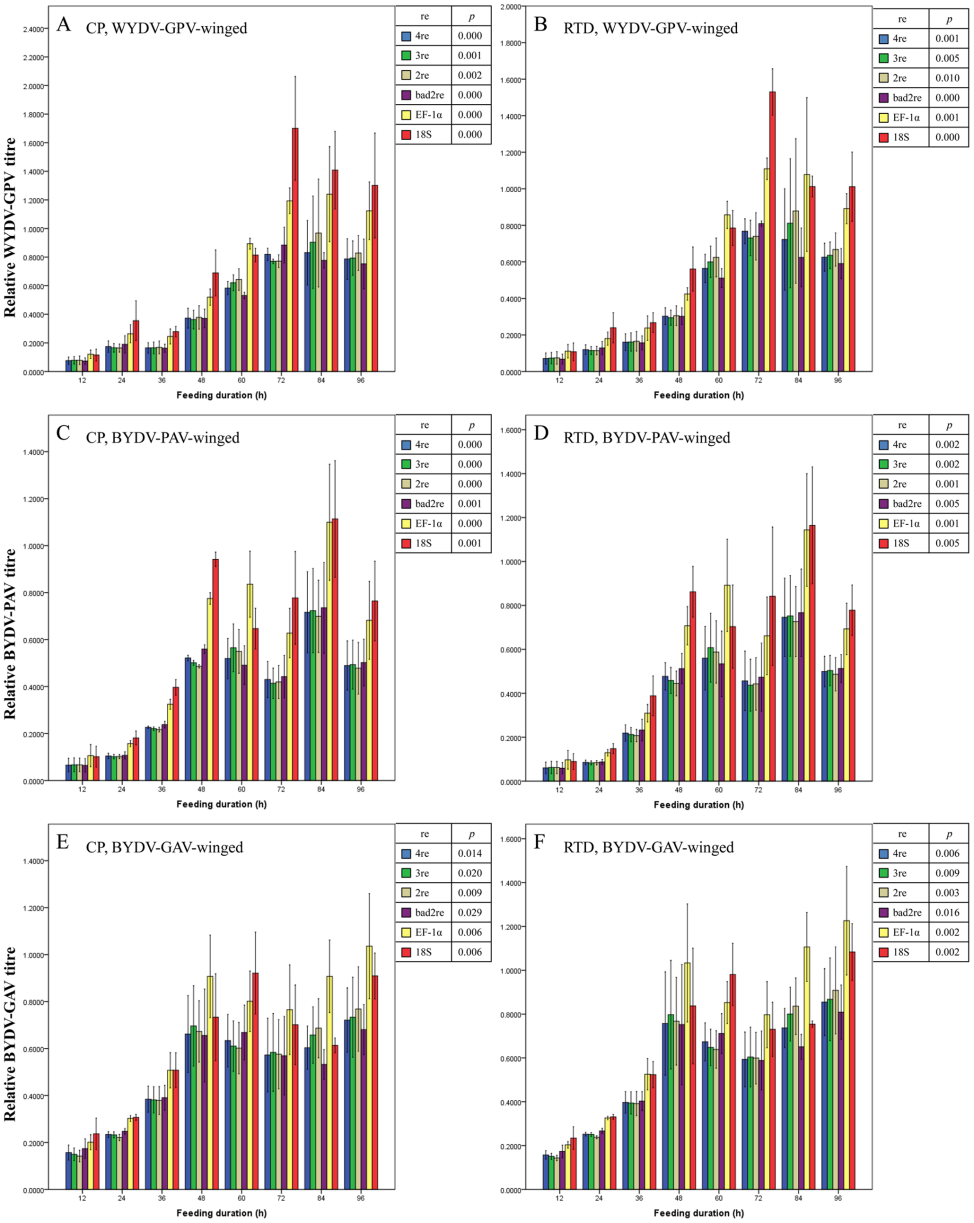
Mean relative titre of YDVs (± SE, *n* = 3) in viruliferous winged adults of *Rhopalosiphum padi* after different virus-feeding durations. Virus titre is illustrated by relative expression values of the CP (A, C and E) and RTD (B, D and F) gene of YDVs. Expression values for the CP or RTD gene at each duration were normalized with the reference gene(s) selected by GeNorm and then compared with a one-way ANOVA (*p*) among these durations with each normalization condition. A and B: WYDV-GPV; C and D: BYDV-PAV; E and F: BYDV-GAV. 2re = 2 best reference genes; 3re = 3 best reference genes; 4re = all 4 reference genes; bad2re = 2 least stable reference genes; EF-1α = EF-1α as the reference gene; 18S = 18S rRNA as the reference gene.

**Figure 10:**
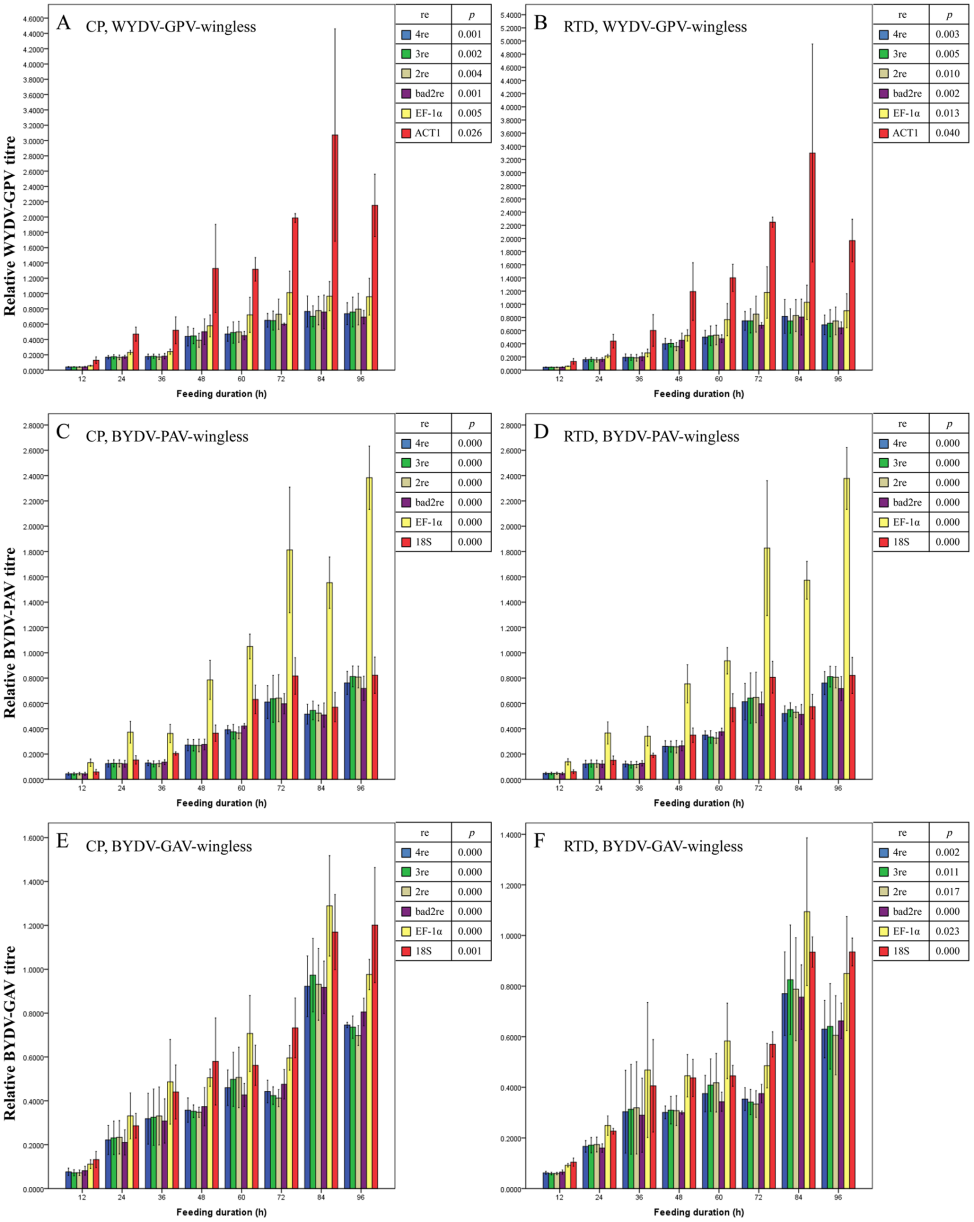
Mean relative titre of YDVs (± SE, *n = *3) in viruliferous wingless adults of *Rhopalosiphum padi* after different virus-feeding durations. Virus titre is illustrated by relative expression values of the CP (A, C and E) and RTD (B, D and F) gene of YDVs. Expression values for the CP or RTD gene at each duration were normalized with the reference gene(s) selected by GeNorm and then compared with a one-way ANOVA (*p*) among these durations with each normalization condition. A and B: WYDV-GPV; C and D: BYDV-PAV; E and F: BYDV-GAV. 2re = 2 best reference genes; 3re = 3 best reference genes; 4re = all 4 reference genes; bad2re = 2 least stable reference genes; EF-1α = EF-1α as the reference gene; 18S = 18S rRNA as the reference gene.

The second one is the relative virus load, normalized by one reference gene or a combination of reference genes (with the same normalization as the endogenous genes). The expression levels of the CP gene and RTD gene of WYDV-GPV, BYDV-PAV and BYDV-GAV in winged (Figure 9 A–F) and wingless *R. padi* (Figure 10 A–F) increased more than 5-fold from the extraordinarily low value at the initial 12 h to the highest value during the last day (72 to 96 h), and the difference in expression of either the CP or RTD gene across all feeding durations was significant (all *p*<0.05, Figures 9 A–F and 10 A–F), regardless of the normalization setting adopted. When the duration of feeding on either WYDV-GPV-, BYDV-PAV- or BYDV-GAV-infected oat plants exceeded 48 h, the relative expression values of the CP gene and RTD gene continued to rise at a slow rate and rose and fell within a narrow range. The findings reflected accumulation but no tendency for replication of the YDV particles in *R. padi* and the saturability of virus load by aphid, in agreement with Gray and Gildow’s hypothesis about the nonpropagative transmission of YDVs by aphids [9]. As shown in Figures 9 and 10, the fluctuation in virus titre of the three viruses in winged and wingless *R. padi* at each duration within the 4-day AAP had some slight differences, relied on the method of normalization, and a good reference gene or reference gene combination estimated by GeNorm resulted in a much lower mean SE value for the sample replicates.

We could not uncover differences in transmissibility of these three YDVs by *R. padi* by comparing the relative virus titre during the first 2 days of feeding with a one-way ANOVA because the relative virus titre between transmissible WYDV-PAV or BYDV-PAV and non- transmissible BYDV-GAV did not differ significantly) (all *p*>0.05, data not shown), even though the relative titre of WYDV-GPV and BYDV-PAV (Figures 9 A–D and 10 A–D) for the 12, 24 and 36 h of feeding was lower than that of BYDV-GAV (Figures 9E, 9F, 10E, and 10F). Perhaps the typical biological transmission assay is the most efficient and convincing measure for determining the insect vector’s transmission specificity for a particular plant virus [19].

With the extension of aphid feeding duration on infected oat plants, the relative expression values of the CP or RTD gene of all three YDVs in *R. padi* increased gradually from 12 h to 60 h or 72 h, but tended to stabilized after 60 h or 72 h. BYDV-PAV and WYDV-GPV once acquired are retained life of *R. padi*. Following ingestion during feeding, the virus moves through the alimentary canal of *R. padi*, invades the gut epithelial cells and then is released into the hemolymph, ultimately infects the salivary glands and is released into the salivary ducts where it can be transferred to new plants via the saliva released during feeding [57]. Virus titre continues to rise during the first 48 h of the AAP and IAP, but decreases slightly because the virus particles are transmitted into plants. As aphids continue sucking the virus-containing phloem sap, the virus load in aphids rises again. For the untransmissible one, the virus only moves through the alimentary canal and then direct excretes from body. Therefore, the virus titre increases at initial feeding duration, and then keeps a dynamic balance because of the continuous sucking [12], [58].

### Conclusions

In this study, we found that in testing EF-1α, ACT1, GAPDH and 18S rRNA as reference genes, combining the first 2 or 3 genes or using all 4 genes can be used to normalize the relative expression level of endogenous and virus titre in BYDV-PAV and BYDV-GAV-viruliferous winged and wingless *R. padi*. Using only one reference gene, regardless of its expression stability, is not recommended for relative quantification by RT-qPCR. Importantly, for accurate and precise results, the use of different regions of a gene, including the 5′-terminus, middle and 3′-terminus, at the same in the relative RT-qPCR is strongly advised, since the different regions of one gene endure different selection pressures and may evolve some other new functions in complex life cycle of aphids. To assess relative expression and virus load in winged and wingless WYDV-GPV-viruliferous *R. padi*, the optimal number of combination of reference genes should be selected carefully, abandoning genes that are not useful alone or in combination and testing new ones as needed.

## Materials and Methods

### Virus Maintenance and Aphid Rearing

Laboratory isolates of WYDV-GPV, BYDV-PAV, and BYDV-GAV have been maintained on oat plants (*Avena sativa* K. Koch cv. Coast-Black Oat) in our laboratory since the 1990s [59]. A virus-free laboratory population of *R. padi* was reared on winter wheat (*Triticum aestivum* L., cv. Yangmai 158), under controlled conditions at 18–23°C. A low-density population was maintained for the wingless morph of *R. padi*, and a high-density population was maintained to obtain the winged morph. This *R. padi* population transmits BYDV-PAV (70–80% transmission rate) and WYDV-GPV (50% transmission rate) effectively, but poorly transmits BYDV-GAV (5–7% transmission rate) [60].

To confirm the morph features of wingless aphids and winged aphids, we observed different developmental stages of *R. padi* from first to fifth instar daily with a stereomicroscope (Olympus SZX16). Because the lifespan of the winged and wingless adult aphids (more than 5 days) is much longer than that of each nymph instar (less than 2 days) and the adult aphids are distinguishable from the nymphs, winged and wingless adults were used for the experiments.

### Experimental Treatment and Sampling

Approximately 300 wingless and 300 winged adult aphids were picked from wheat with a soft brush, and then released carefully onto two oat plants infected with WYDV-GPV, BYDV-PAV or BYDV-GAV for acquisition of viruses, with insect-proof nets separating the three sets of infected plants. After each feeding duration (0, 12, 24, 36, 48, 60, 72, 84 or 96 h), 24 wingless adult aphids in each net were collected and divided randomly into 3 pools, then 8 aphids of each type were placed into an RNase-free tube. The winged adult aphids were treated the same way. The tubes were immediately placed in liquid nitrogen and stored in a −70°C freezer for RNA extraction.

### Selection of Candidate Reference Genes and Target Genes and Design of Primers

Four HKGs of *R. padi*, including 18S rRNA, ACT1, EF-1α, and GAPDH were selected as candidate reference genes (Table 7). Based on the corresponding sequences of *R. padi* and *A. pisum* from the NCBI GenBank, primers for reverse transcription-PCR (RT-PCR) amplification of 18S rRNA and EF-1α were designed (Table 1). Primer pairs for amplifying the ACT1 and GAPDH sequences of *R. padi* were designed using homologous sequences of *A. pisum* deposited in GenBank (Table 1).

**Table 7:** Description of candidate reference genes for *Rhopalosiphum padi* and of target genes.

Genes		Gene name	Molecular function^a^		Source^b^		
Candidate referencegenes							
	18S rRNA	18S ribosomal RNA (18S rRNA)		Participates in protein translationof Eukaryotes		U27825	
	ACT1	Actin1		Cytoskeletal structural protein		NM_001126200	
	EF-1α	Elongation factor-1 alpha		Involved in protein biosynthesis		AY219719	
	GAPDH	Glyceraldehyde-3-phosphatedehydrogenase		Catalyzes the reversible oxidativephosphorylation		XM_001943014	
Endogenous genes							
	*ago-1a*-1	Argonaute-1 region1		miRNA machinery; RISCkey component		HE585880-81	
	*ago-1a*-2	Argonaute-1 region2		miRNA machinery; RISCkey component		HE585908-09	
	*ago-1a*-3	Argonaute-1 region3		miRNA machinery; RISCkey component		HE585932-33	
	*dcr1*	Dicer-1		RNase III enzyme		HE585957-58	
Viral genes							
	BYDV-GPV CP	*Polerovirus* Wheat yellowdwarf virus-GPV, Coatprotein		Encodes major structuralprotein of virus particle; aphidtransmission-related protein		FM865413	
	BYDV-GPV RTD	*Polerovirus* Wheat yellowdwarf virus-GPV,Readthrough domain		Encodes minor structuralprotein of virus particle; aphidtransmission-related protein		FM865413	
	BYDV-PAV CP	*Luteovirus* Barley yellowdwarf virus-PAV, Coatprotein		Encodes major structuralprotein of virus particle; aphidtransmission-related protein		EU332307-336	
	BYDV-PAV RTD	*Luteovirus* Barley yellowdwarf virus-PAV,Readthrough domain		Encodes minor structuralprotein of virus particle; aphidtransmission-related protein		EU332307-386	
	BYDV-GAV CP	*Luteovirus* Barley yellowdwarf virus-GAV, Coatprotein		Encodes major structuralprotein of virus particle; aphidtransmission-related protein		EU402386-391	
	BYDV-GAV RTD	*Luteovirus* Barley yellowdwarf virus-GAV,Readthrough domain		Encodes minor structuralprotein of virus particle; aphidtransmission-related protein		EU402386-391	

a Terminology cited from Gene Ontology (GO) of NCBI.

b GenBank accession number (NCBI).

Two endogenous genes of *R. padi*, *ago-1a* and *dcr1*, which might be involved in the miRNA pathway in aphids [48], [49], were used as target genes. The 3 packed regions of the *ago-1a* have been showed different selective pressures, region 1 and region 2 reflecting strong purifying selection (negative selection), but region 3 owning no positively selected sites [49]. The chosen region of *dcr1* has the same selection pressure with region 3 of *ago-1a* [49]. For the RT-PCR of these two genes, primers (Table 1) were devised using the DNA regions for the *ago-1a* gene and *dcr1* gene.

The CP gene (ORF3), encoding the coat protein, and RTD gene (ORF5), encoding the readthrough protein, of WYDV-GPV, BYDV-PAV and BYDV-GAV from infected oat plants were amplified by RT-PCR using their respective primers (Table 1). All primers used in this study were synthesized by Sangon Biotech (Shanghai) Co.

### RNA Extraction and Purification

For plant leaves and a large number of aphids, samples were first homogenized in liquid nitrogen with a sterile mortar and pestle. Then total RNA was extracted from the resulting fine powder using the instructions for TRIzol reagent (Ambion, USA) with minor modifications. RNA was precipitated in isopropanol overnight at 4°C. The 8-aphid pooled sample was frozen with liquid nitrogen and ground with a clean glass rod for total RNA extraction in 400 µl TRIzol reagent. The concentration and purity of RNA samples were measured with a NanoDrop-2000 spectrophotometer (Thermo Fisher Scientific, Roskilde, Denmark). The integrity was checked using electrophoresis in 1.5% agarose gels and 1× Tris-acetate EDTA (TAE). The gel was then photographed in UV light (Bio-Rad, Universal Hood II, USA).

### RT-PCR of Candidate Reference and Target Genes

The total RNA isolated from plant leaves and the aphid samples of all developmental stages was reverse transcribed into first-strand cDNA only using the reverse primer of each gene. The components in each 25-µl RT reaction system included 7 µl RNase-free ddH_2_O, 1 µl 5 µM reverse primer and 2 µl total RNA. The mixture was briefly centrifuged and denatured at 70°C for 10 min, and then combined with 3.5 µl RNase-free ddH_2_O, 5 µl dNTPs (2.5 µM, TaKaRa, Dalian, China), 5 µl 5× RT reaction buffer, 1 µl M-MLV Reverse Transcriptase (200 U/µl, Promega, Madison, WI, USA), 0.5 µl Recombinant RNase Inhibitor (40 U/µl, TaKara). All reagents in the tube were centrifuged shortly and incubated at 42°C for 1 h. The RT reaction was terminated at 95°C for 5 min. The final cDNA samples were kept at −20°C.

The PCR reaction mixture for each first-strand cDNA comprised 17.8 µl ddH_2_O, 0.25 µl forward primer (5 µM), 0.25 µl reverse primer (5 µM), 10× *LA Taq* Buffer II (Mg^2+^ plus), 2 µl dNTPs, and 0.2 µl *LA Taq* (TaKaRa). The PCR program for all reactions was set as follows: pre-denaturation at 94°C for 4 min; 32 cycles of denaturation at 94°C for 1 min, annealing at 56–70°C for 1 min 30 s, and elongation at 72°C for 1 min 30 s; and 10 min at 72°C for the final elongation. RT-PCR products were purified and ligated with pMD18T simple vector (TaKaRa), and finally transformed into competent DH5α *E. coli* cells (TIANGEN BIOTECH, Beijing, China) according to a standard transformation method [61]. Three to five positive clones were sequenced by Sangon Biotech (Shanghai) Co., Ltd.

### RT-qPCR Data Collection

Two-step RT-qPCR was used to obtain quantification cycle values (Cq, terminology from MIQE [62]) for all genes of all samples. First-stranded cDNA of each sample was synthesized according to the protocol of FastQuant RT Kit (with gDNase, TIANGEN BIOTECH). Genomic DNA (gDNA) in a 20 µl RT reaction system was first removed by adding 2 µl 5× gDNA Buffer, 1 µg total RNA, and appropriate RNase-Free ddH_2_O, and incubating at 42°C for 3 min. Then 2 µl 10× Fast RT Buffer, 2 µl FQ-RT Primer Mix (random hexamers and oligo-dT primers), 1 µl RT Enzyme Mix, and 5 µl RNase-Free ddH_2_O were added and the mixture held at 42°C for 15 min, then at 95°C for 3 min. The final cDNA was kept on ice or at −20°C for the qPCR. Subsequently, SYBR Green I dye-based qPCR was performed for all cDNA samples. Each 20 µl qPCR reaction system contained 6.4 µl RNase-free ddH_2_O, 10 µl 2× SuperReal PreMix Plus (SuperReal PreMix Plus kit [SYBR Green I, TIANGEN BIOTECH]), 0.6 µl forward primer (10 µM), 0.6 µl reverse primer (10 µM), 0.4 µl 50× ROX Reference Dye, 2 µl diluted cDNA (1∶30 diluted cDNA sample template) or ddH_2_O (as the no-template control). qPCRs of all genes were run on a 7500 real time PCR system (Applied Biosystems) with the same program: pre-denaturation at 95°C for 15 min, and 3-step amplification procedure of 40 cycles of 95°C for 10 s, 59.2°C for 32 s and 72°C for 32 s; followed by the melting curve stage of 95°C for 15 s, 60°C for 1 min, 95°C for 30 s and 60°C for 15 s. The primers used for the qPCR reactions are listed in Table 2. In each single run, the raw Cq values were automatically calculated by the 7500 real time PCR system detection software. Three replicate qPCRs were done for each cDNA dilution sample. The amplification efficiency (*E*) of each candidate reference gene and the endogenous genes were calculated using the Cq values for a 5-fold dilution series of the cDNA (1∶5, 1∶25, 1∶125, 1∶625, 1∶3,125, and 1∶15,625). For virus genes, *E* values were determined using a 2-fold dilution series of cDNA (1∶2, 1∶4, 1∶8, 1∶16, 1∶32, and 1∶64).

### Sequence Analysis

The nucleotide sequences of all genes that were cloned from the cDNA were analyzed using the BLAST tool on the NCBI website (http://blast.ncbi.nlm.nih.gov/) to confirm the genes. Vector NTI Advance 10.3 package (Invitrogen) served as the alignment tool.

### Algorithms and Statistical Analysis

Expression stability of 4 candidate reference genes in YDV-viruliferous *R. padi* after the AAPs was evaluated by three algorithms: NormFinder 0.953 [37], BestKeeper version1 [38], and GeNorm3.5 [25] respectively. Cq values were analyzed directly with BestKeeper, and then the stability of the HKGs was ordered according to standard deviation (SD) values. In addition, linear scale expression quantities (*Q*) were transformed from the Cq values by the delta-Cq method (ΔCq = min Cq − sample Cq; min Cq = lowest Cq value of the data set, sample Cq = Cq values of other samples) and corrected by the modified amplification efficiency (*E*′ = *E* +1) for the individual gene, according to the formula *Q* = *E*′ ^ΔCq^, then analyzed by NormFinder and GeNorm.

To reach a reliable and unambiguous conclusion about qPCR results, we need two or more reference genes that are stably, i.e., consistently, expressed to normalize calculations [25], [62]. In addition to ranking the expression stability of a gene, GeNorm can also determine the optimal number of reference genes by pairwise variation, *V*, between two sequential normalization factors containing an increasing number of reference genes, whose default cut-off value of 0.15 has been accepted by many studies of finding reference genes [15], [44], [63]. When values are higher than 0.15, representing a large variation between the two sequential normalization factors, then another reference gene needs to be included to calculate a dependable normalization factor [25]. The pairwise variation starts from the first two and three (*V*2/3) most stable candidate reference genes, terminating with the addition of the most unstable one. As the number of reference genes increases, the best number (*n*) of reference genes for calculating the normalization factor will be decided, because the value of *Vn*/*n* +1 being first lower than 0.15, which means the variation in normalization factors from between *n* and *n* +1 reference genes is so small that an additional reference gene does not need to be included for calculating the normalization factor [25].

The normalization factors produced by GeNorm for the selected reference genes were used to calculate the relative virus titre and the expression value for the endogenous genes (as described in the GeNorm manual). To reach a persuasive conclusion, we used the best single reference gene (EF-1α for all experimental groups), the worst single reference gene (ACT1 for WYDV-GPV-wingless group, and 18S rRNA for the remaining 5 groups), a 4-reference gene combination (4re), 3-reference gene combination (3re), 2 best reference genes (2re) and 2 worst reference gene combination (bad2re) (according to Figure 2B) to normalize the expression levels for *ago-1a* and *dcr1* in each group (using the serial formulas below ). The means among treatments were compared by a one-way ANOVA.
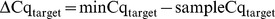
(1)




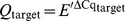
(2)




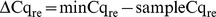
(3)




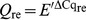
(4)




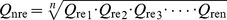
(5)




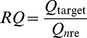
(6)




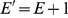
(7)


All statistical analyses were done with the program SPSS version 19 (IBM SPSS Statistics 19 Core System, USA). Plots were graphed using SigmaPlot version 12.5 (Systat, San Jose, CA, USA).

## Supporting Information

Figure S1 **Total RNA extraction of oats and aphids.** (A) Oat RNAs. H: healthy oat plants; BYDV-PAV: BYDV-PAV-infected oat plants; WYDV-GPV: WYDV-GPV-infected oat plants; BYDV-GAV-infected oat plants. (B) RNAs from mixed developmental stages of *Rhopalosiphum padi*; (C) RNAs from BYDV-PAV- winged or wingless adult after various feeding durations (h); (D) RNAs from WYDV-GPV- winged or wingless adult after various feeding durations (h); (E) RNAs from BYDV-GAV- winged or wingless adult after various feeding durations (h). M: DL2000 (TaKaRa).
(PDF)

Figure S2 **RT-PCR amplification of candidate reference genes and target genes.** (A) RT-PCR amplification of the CP and RTD genes from total RNAs of BYDV-PAV-, WYDV-GPV- and BYDV-GAV-infected oat plants in Figure S1 (A). H: healthy oat plants; W: ddH_2_O as no-template control; 1–4: four YDV-infected oat plants; (B) RT-PCR amplification of 4 candidate reference genes from total RNA in Figure S1 (B). Four lanes of each candidate gene indicated four RT-PCR products of 1/100 diluted RNA sample under different annealing temperature; (C) RT-PCR amplification of endogenous genes from total RNA in Figure S1 (B). Eight lanes of each gene indicated eight RT-PCR products of 1/10 (first four lanes) and 1/100 (last four lanes) diluted RNA sample under different annealing temperature. M: DL2000.
(PDF)

Figure S3 **Melt curve and gel photo to check specificity and the size of RT-qPCR primer pair of each gene.** NTC: no-template control; 0 h: 0 h feeding duration; selected samples: 12 randomly selected samples; M: DL2000.
(PDF)

Figure S4 **Relative standard curves to determine amplification efficiencies of candidate reference genes and endogenous genes of the aphid** ***Rhopalosiphum padi*** **(diluted cDNA from virus-free winged and wingless adult aphids) and CP and RTD genes of YDVs (diluted cDNA from YDV-viruliferous winged and wingless aphids after a 12-h virus-feeding.**
(PDF)

Table S1 **Result of best Blast search hit of 18S rRNA, ACT1, EF-1α, and GAPDH.**
(PDF)

Table S2 **BestKeeper analysis of virus titre in YDV-viruliferous winged adults of** ***Rhopalosiphum padi*** **after different virus-feeding durations.** *n* = 24: total number of samples used for analysis; Geo mean: geometric mean; Ar Mean: arithmetic mean; Min: minimun value of Cq; Max: maximum value of Cq; SD: standard deviation; CV: coefficient of variance.
(PDF)

Table S3 **BestKeeper analysis of virus titre in YDV-viruliferous wingless adults of** ***Rhopalosiphum padi*** **after different virus-feeding durations.** *n* = 24: total number of samples used for analysis; Geo mean: geometric mean; Ar Mean: arithmetic mean; Min: minimun value of Cq; Max: maximum value of Cq; SD: standard deviation; CV: coefficient of variance.
(PDF)
