# Supplementary figures and images for: Sequencing and Validation of Reference Genes to Analyze Endogenous Gene Expression and Quantify Yellow Dwarf Viruses Using RT-qPCR in Viruliferous Rhopalosiphum padi
Source: PLoS One. 2014 May 8;9(5):e97038. doi: 10.1371/journal.pone.0097038 (PMC4014588; doi:10.1371/journal.pone.0097038)

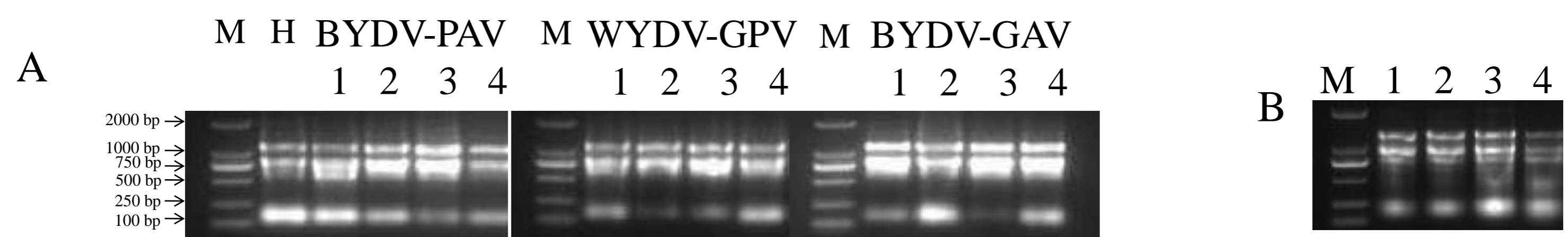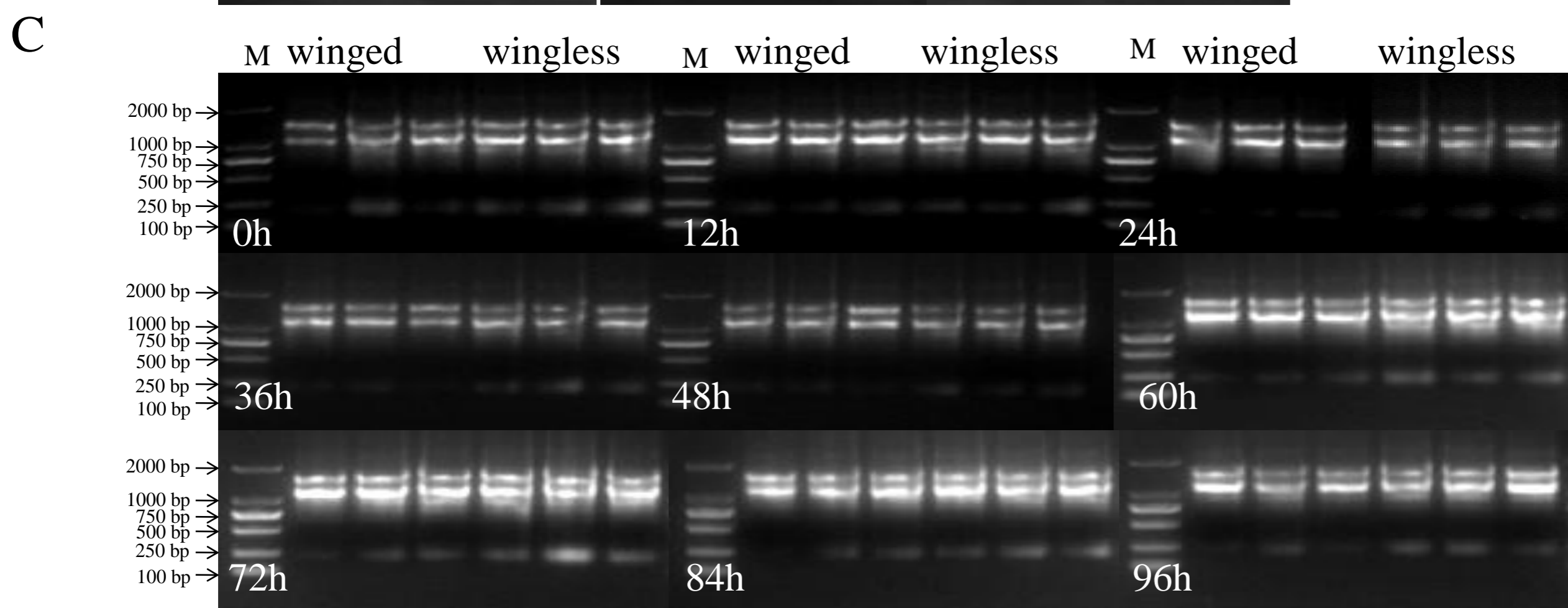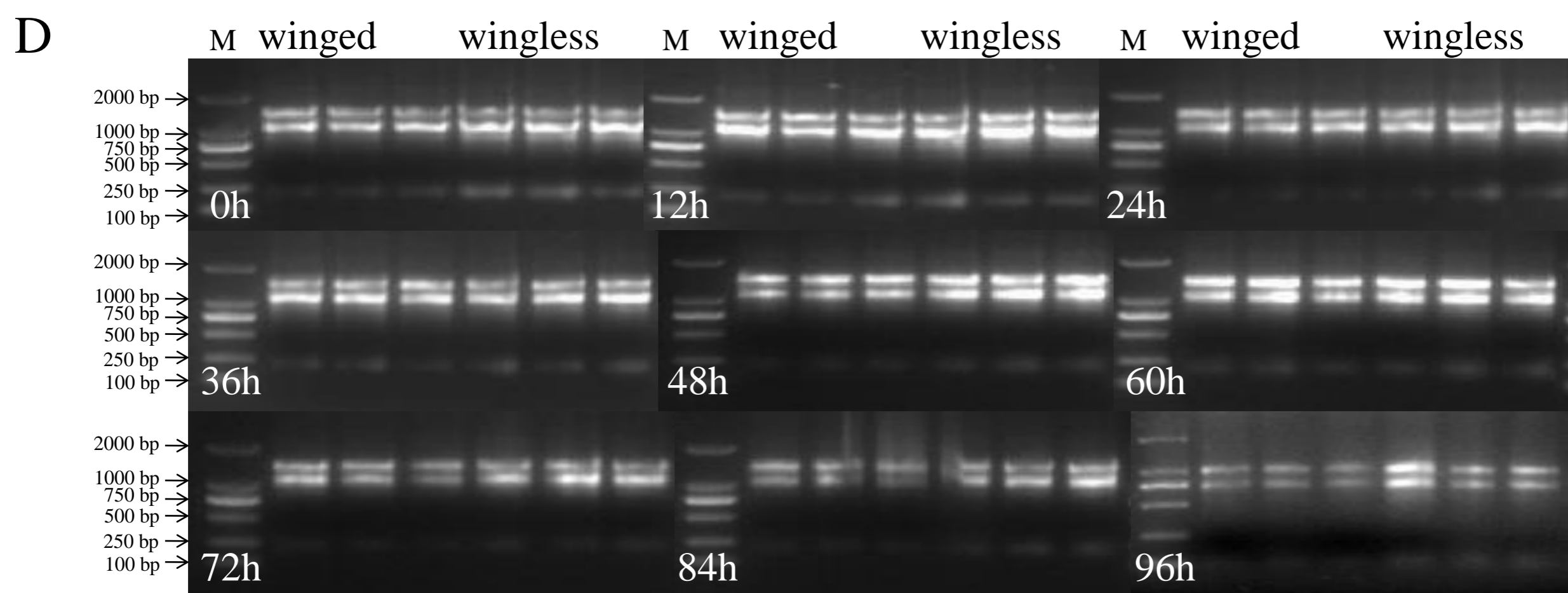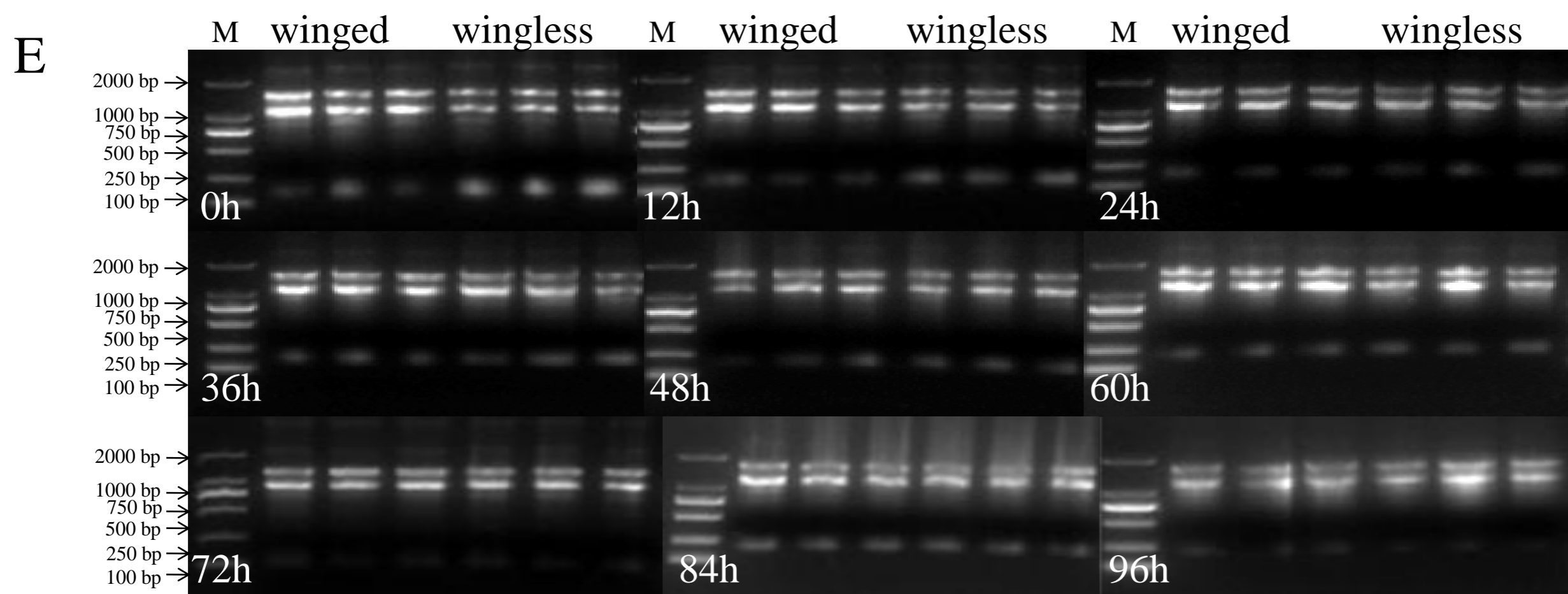

Supplement: Figure S1 — Total RNA extraction of oats and aphids. (A) Oat RNAs. H: healthy oat plants; BYDV-PAV: BYDV-PAV-infected oat plants; WYDV-GPV: WYDV-GPV-infected oat plants; BYDV-GAV-infected oat plants. (B) RNAs from mixed developmental stages of Rhopalosiphum padi; (C) RNAs from BYDV-PAV- winged or wingless adult after various feeding durations (h); (D) RNAs from WYDV-GPV- winged or wingless adult after various feeding durations (h); (E) RNAs from BYDV-GAV- winged or wingless adult after various feeding durations (h). M: DL2000 (TaKaRa). (PDF) [file pone.0097038.s001.pdf]

**A**

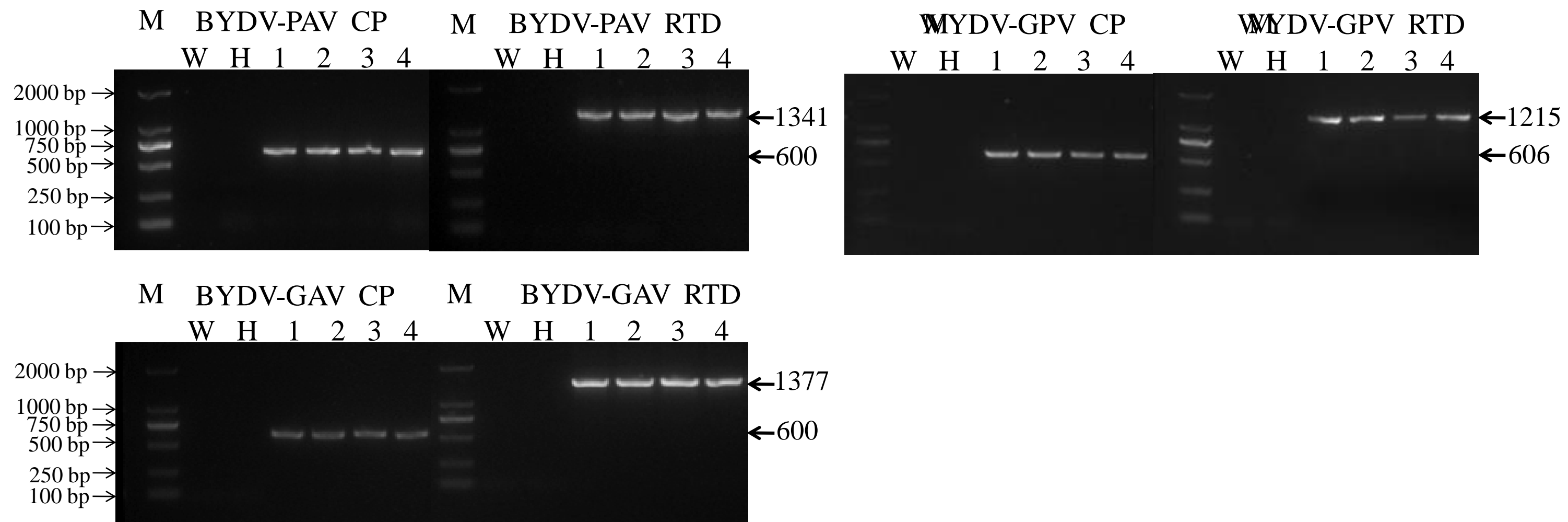

**B**

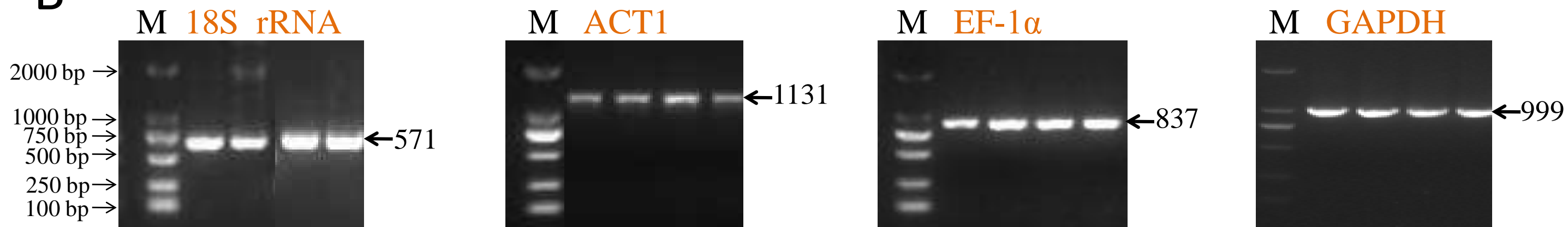

**C**

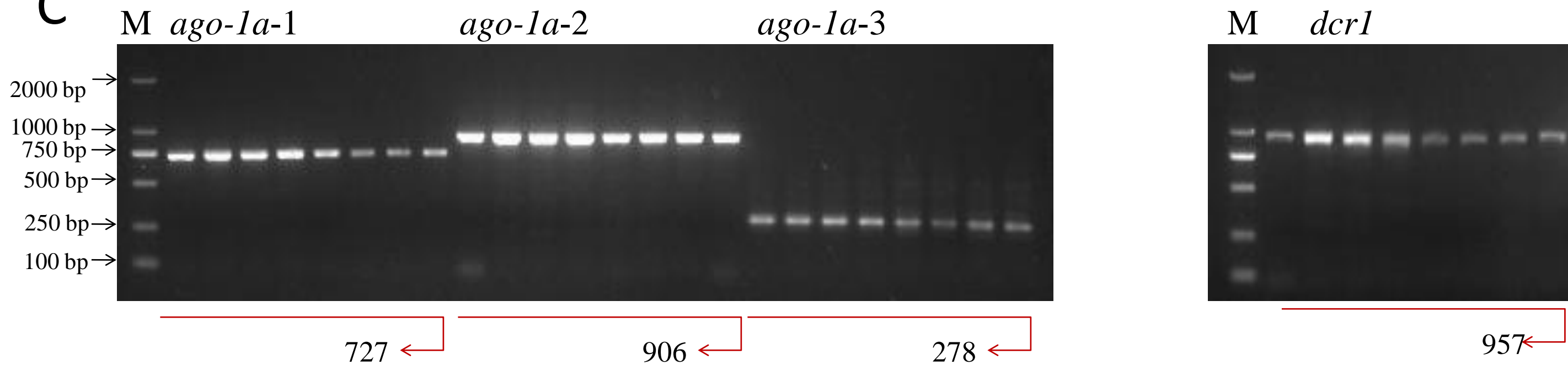

Supplement: Figure S2 — RT-PCR amplification of candidate reference genes and target genes. (A) RT-PCR amplification of the CP and RTD genes from total RNAs of BYDV-PAV-, WYDV-GPV- and BYDV-GAV-infected oat plants in Figure S1 (A). H: healthy oat plants; W: ddH2O as no-template control; 1–4: four YDV-infected oat plants; (B) RT-PCR amplification of 4 candidate reference genes from total RNA in Figure S1 (B). Four lanes of each candidate gene indicated four RT-PCR products of 1/100 diluted RNA sample under different annealing temperature; (C) RT-PCR amplification of endogenous genes from total RNA in Figure S1 (B). Eight lanes of each gene indicated eight RT-PCR products of 1/10 (first four lanes) and 1/100 (last four lanes) diluted RNA sample under different annealing temperature. M: DL2000. (PDF) [file pone.0097038.s002.pdf]

Melt curve and gel photo to check the specificity of RT-qPCR primer pair of each gene

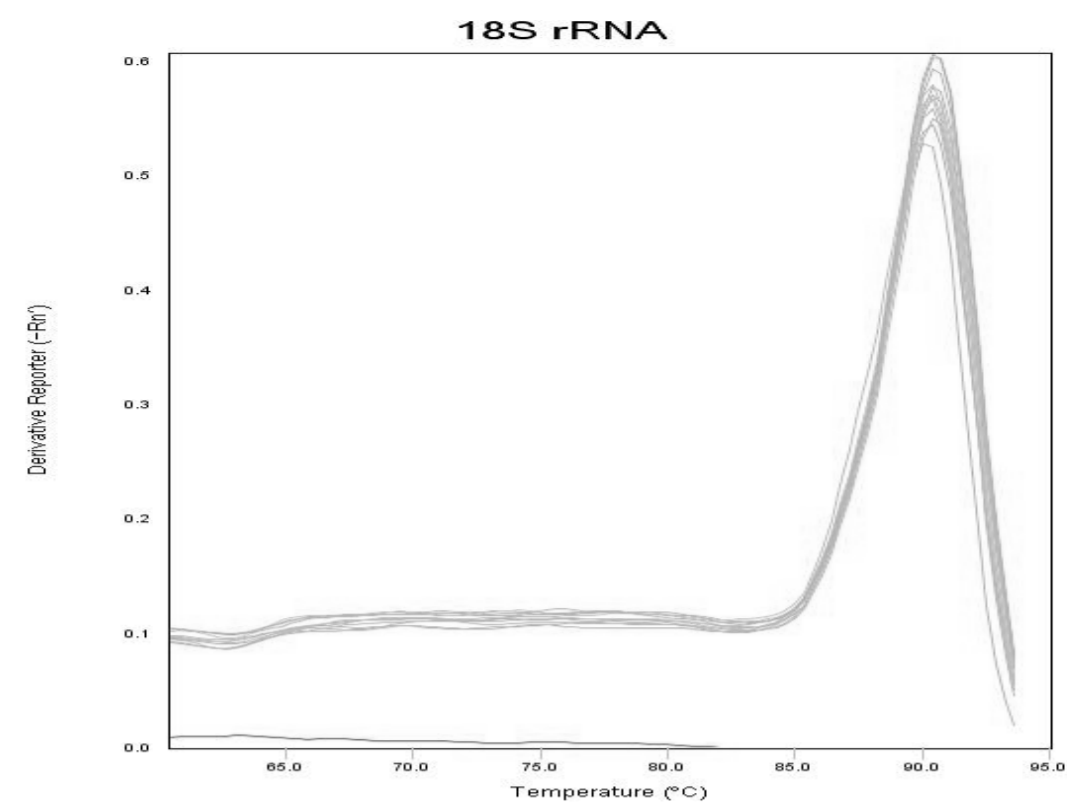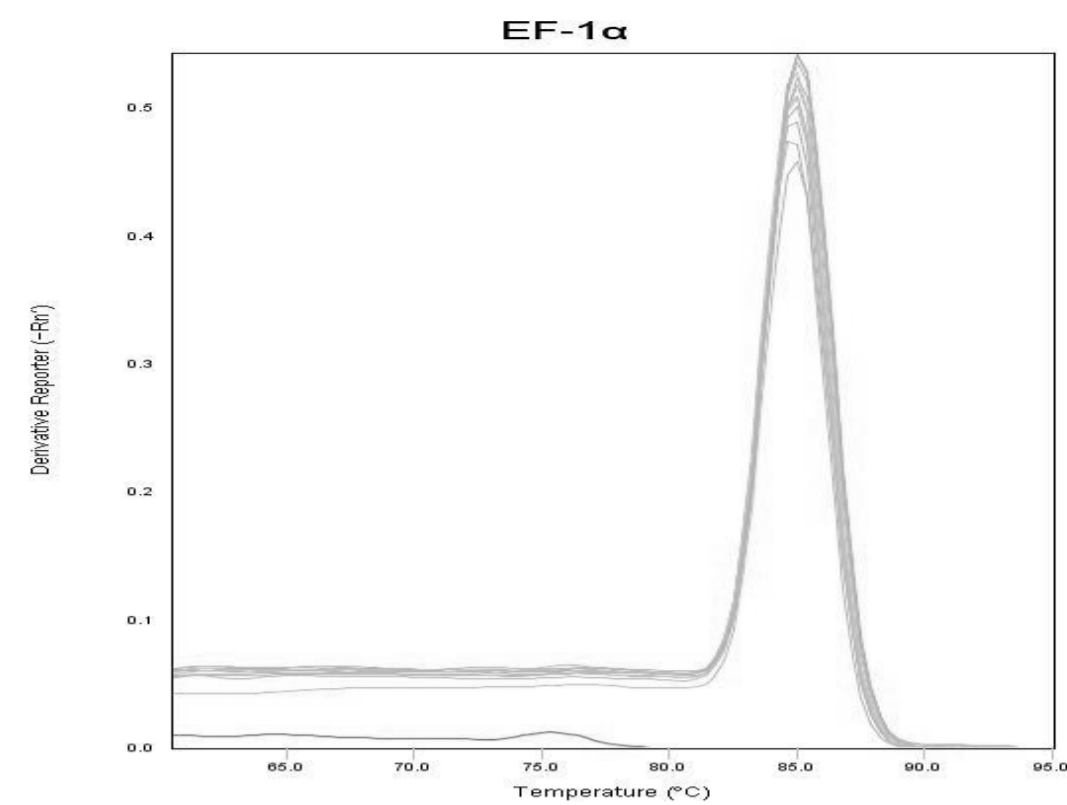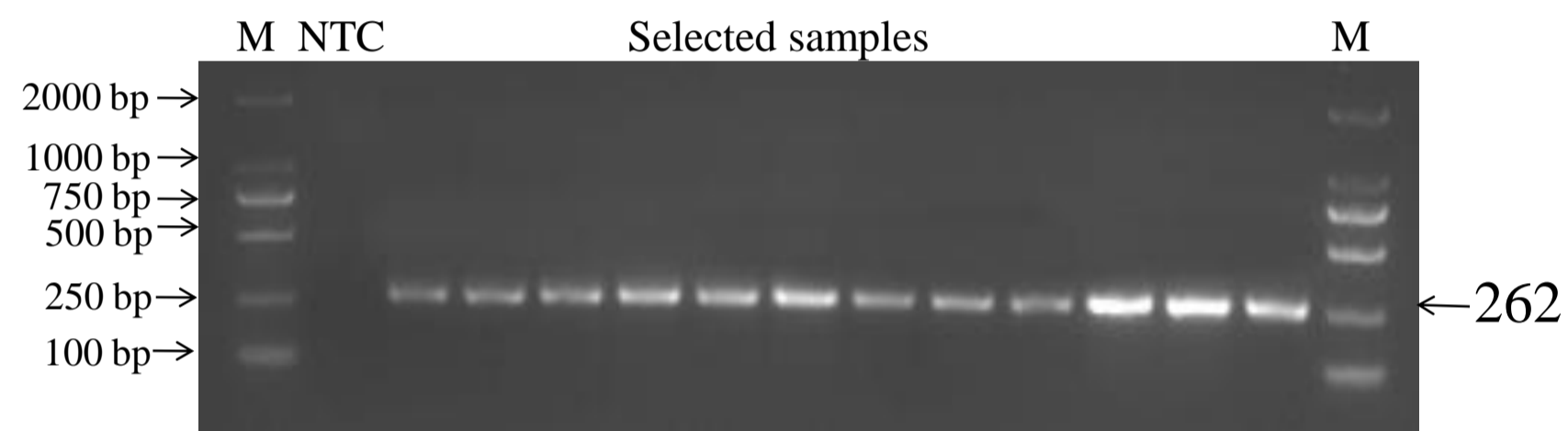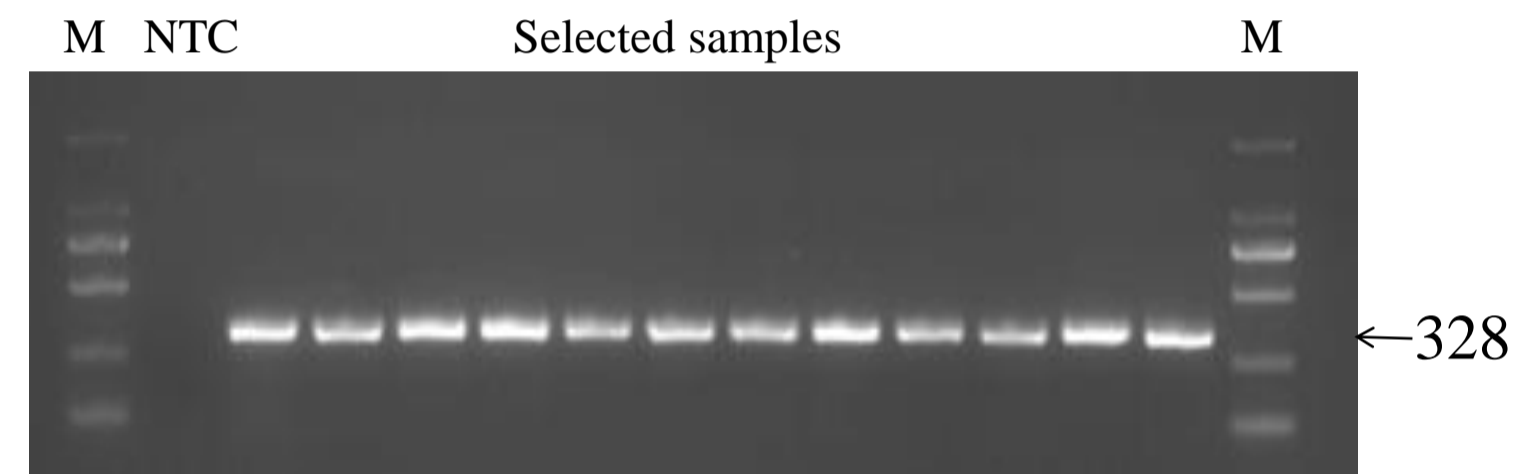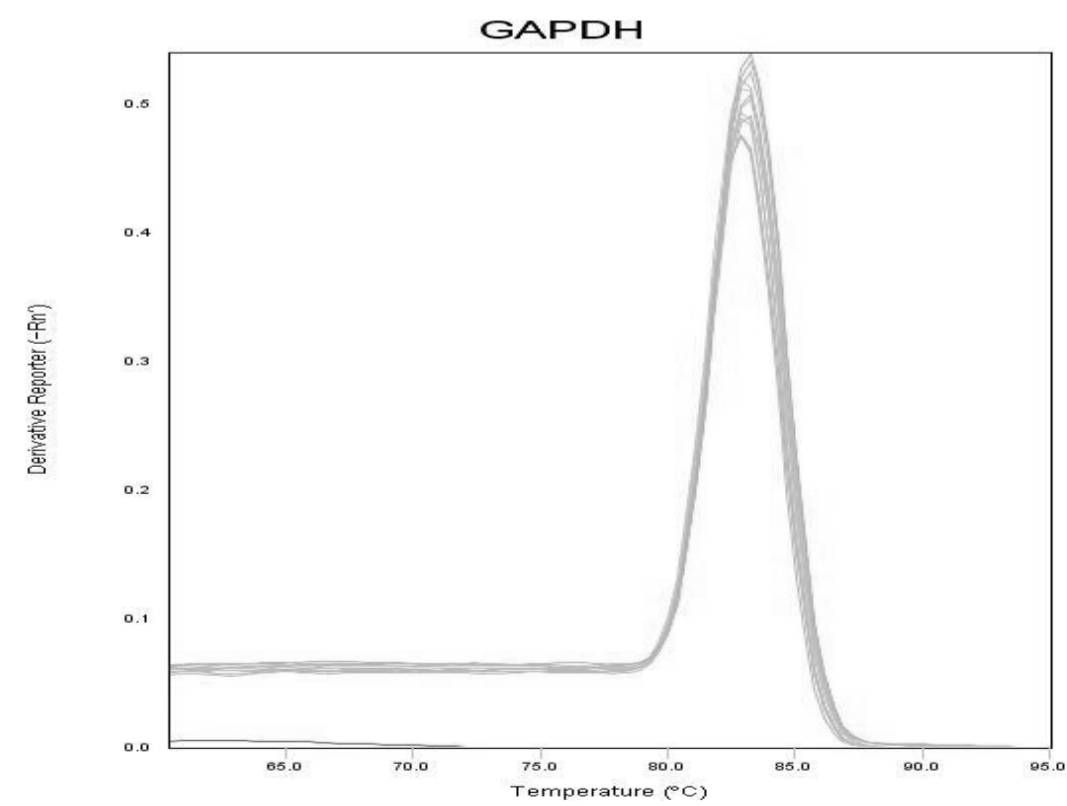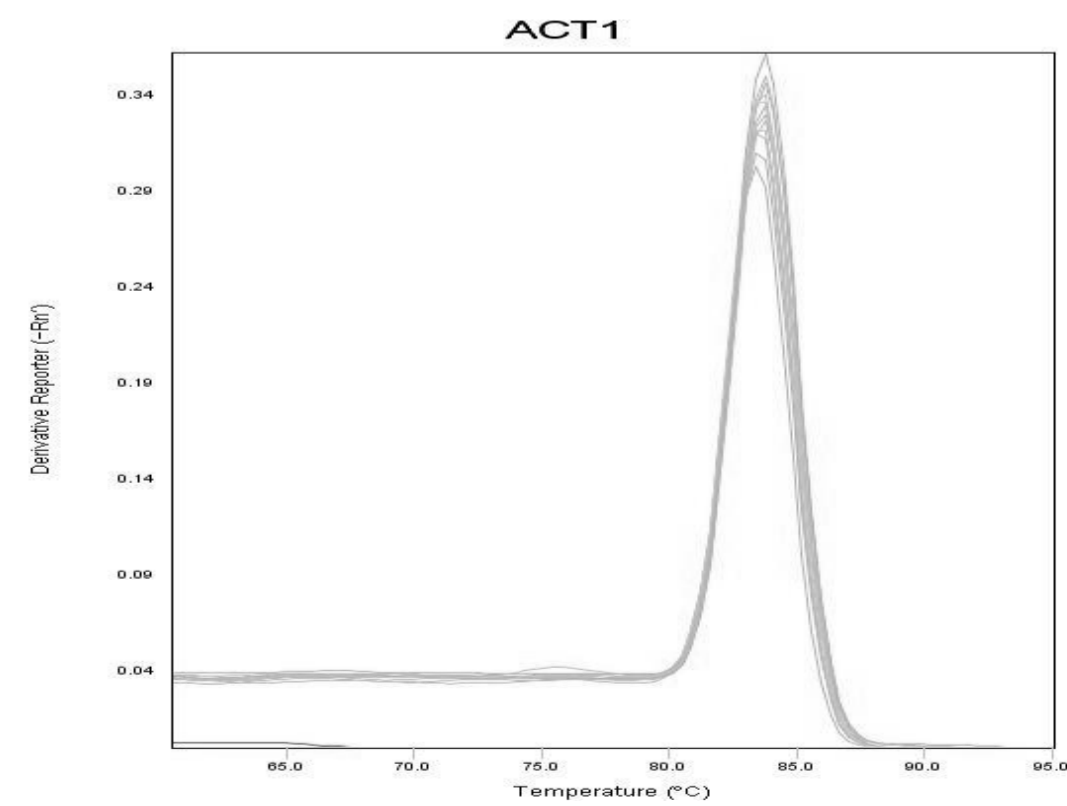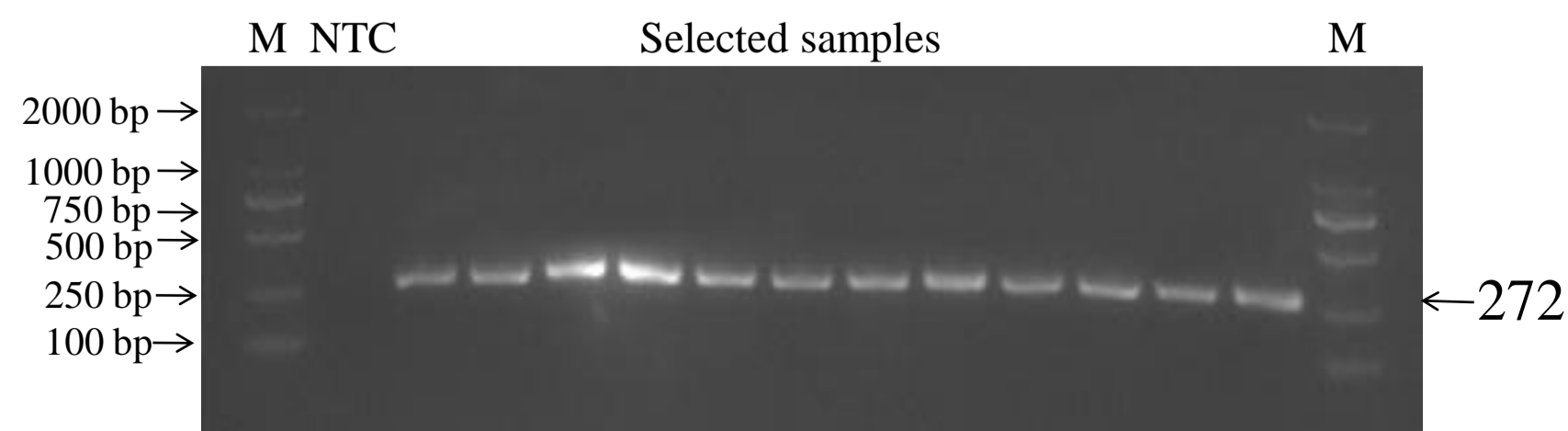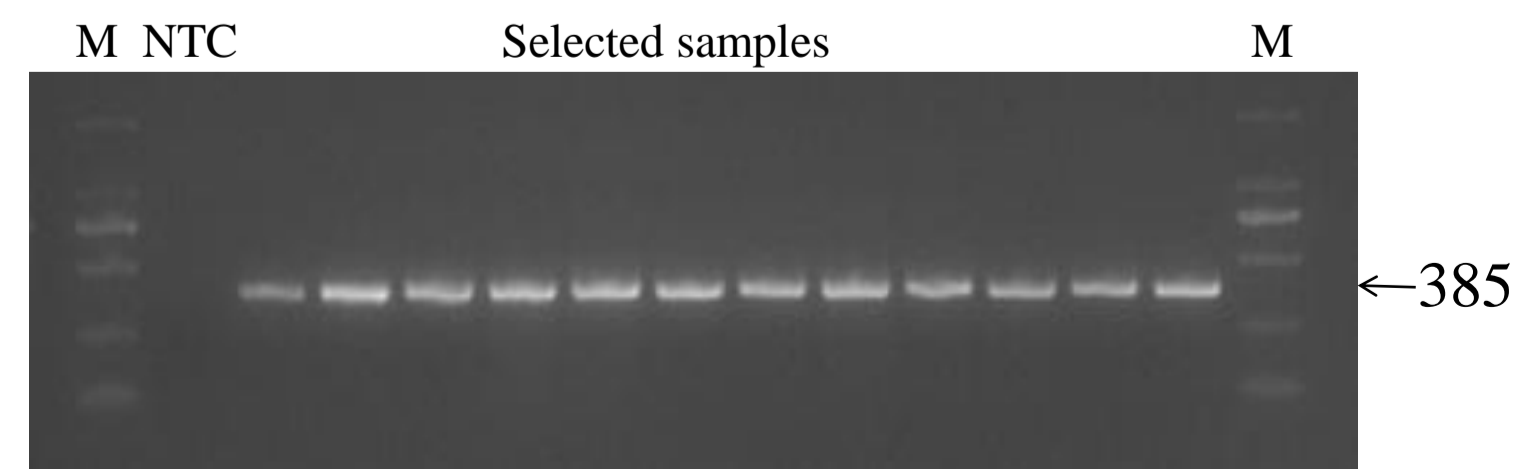

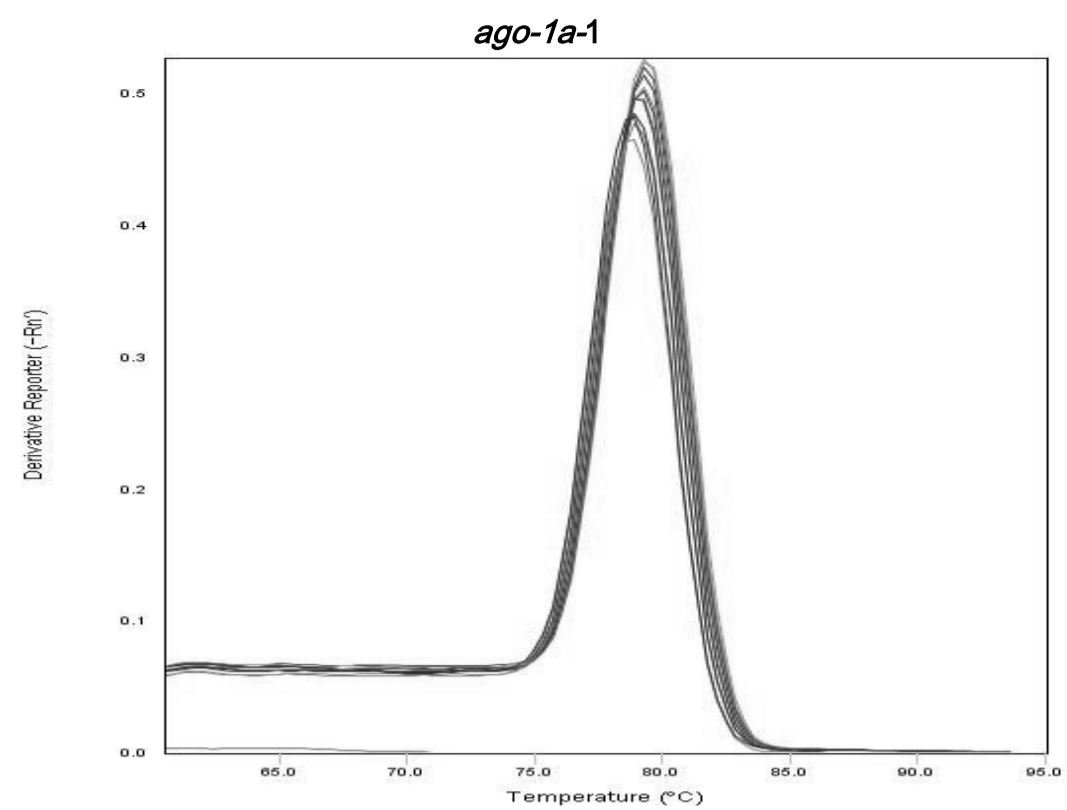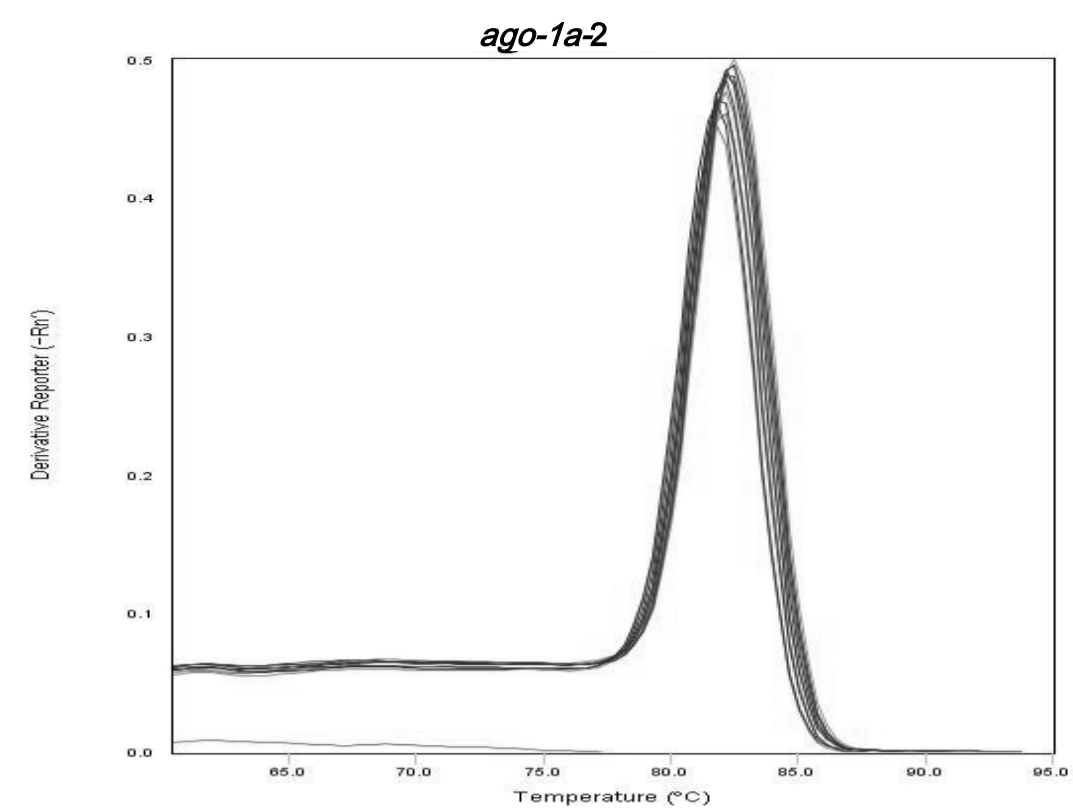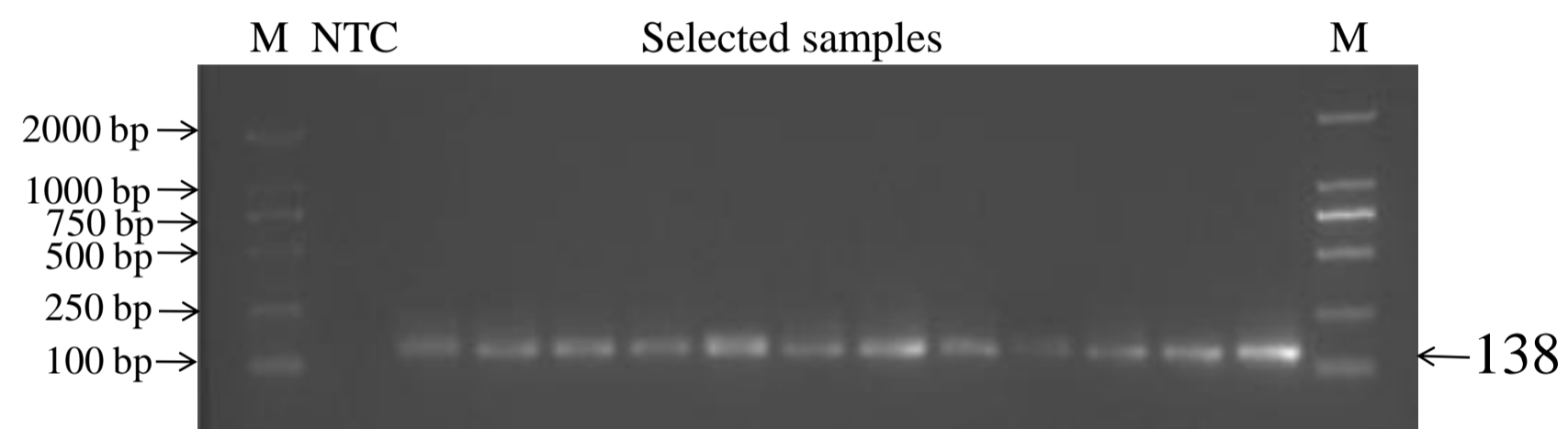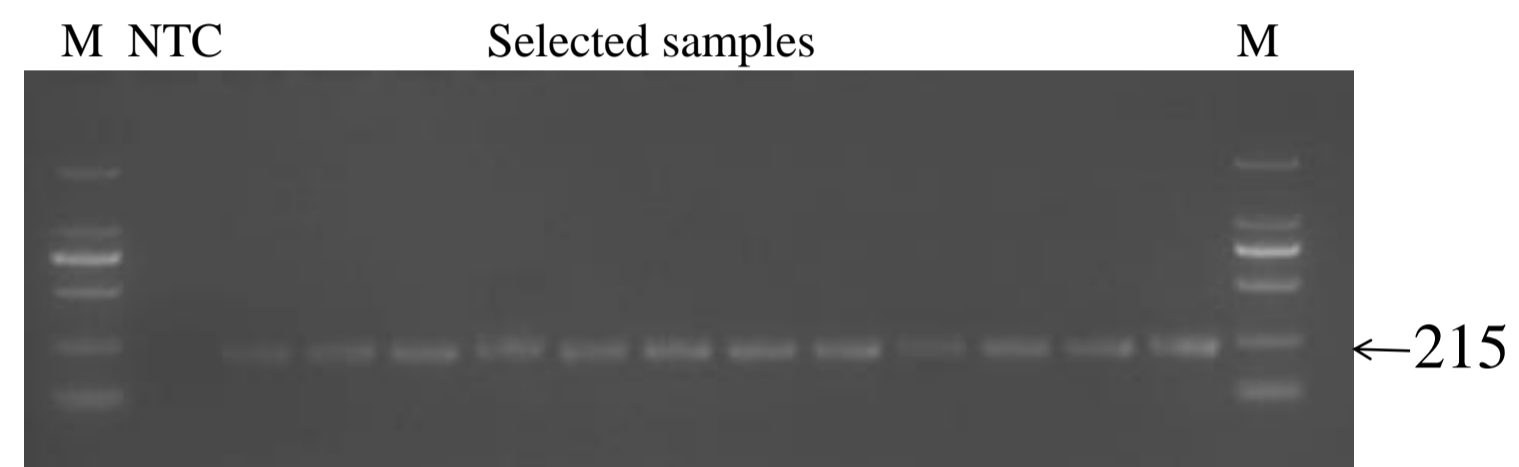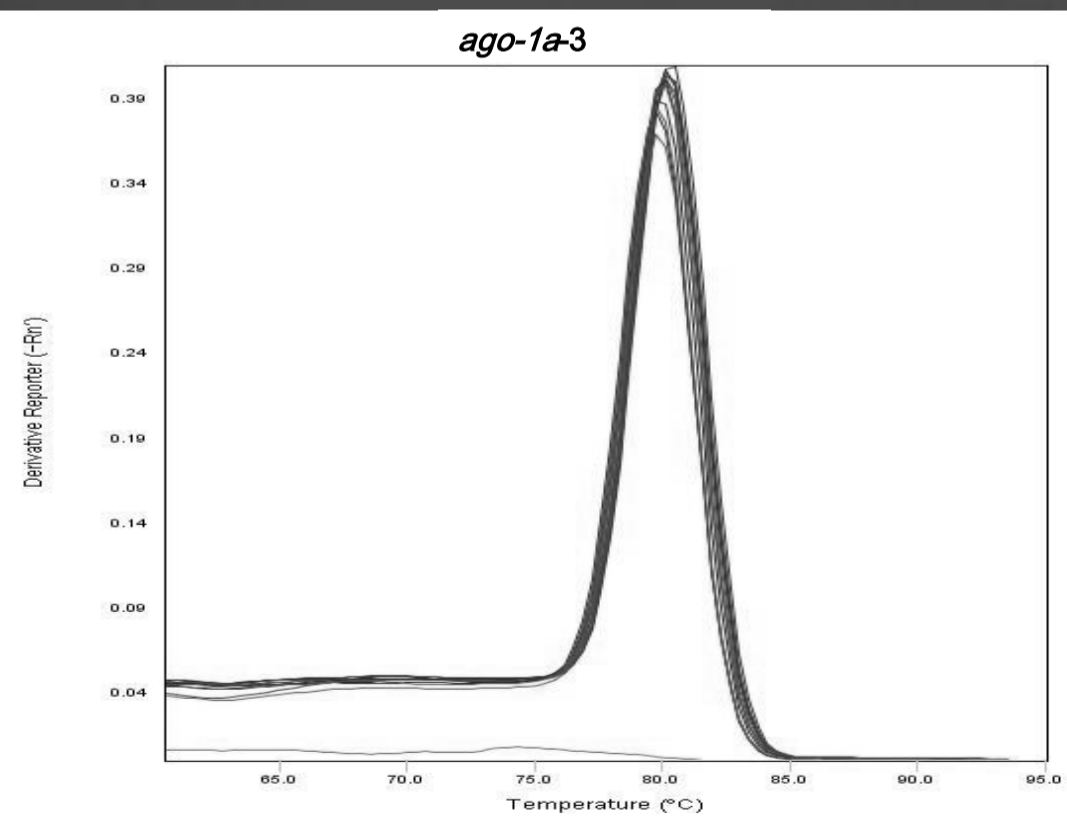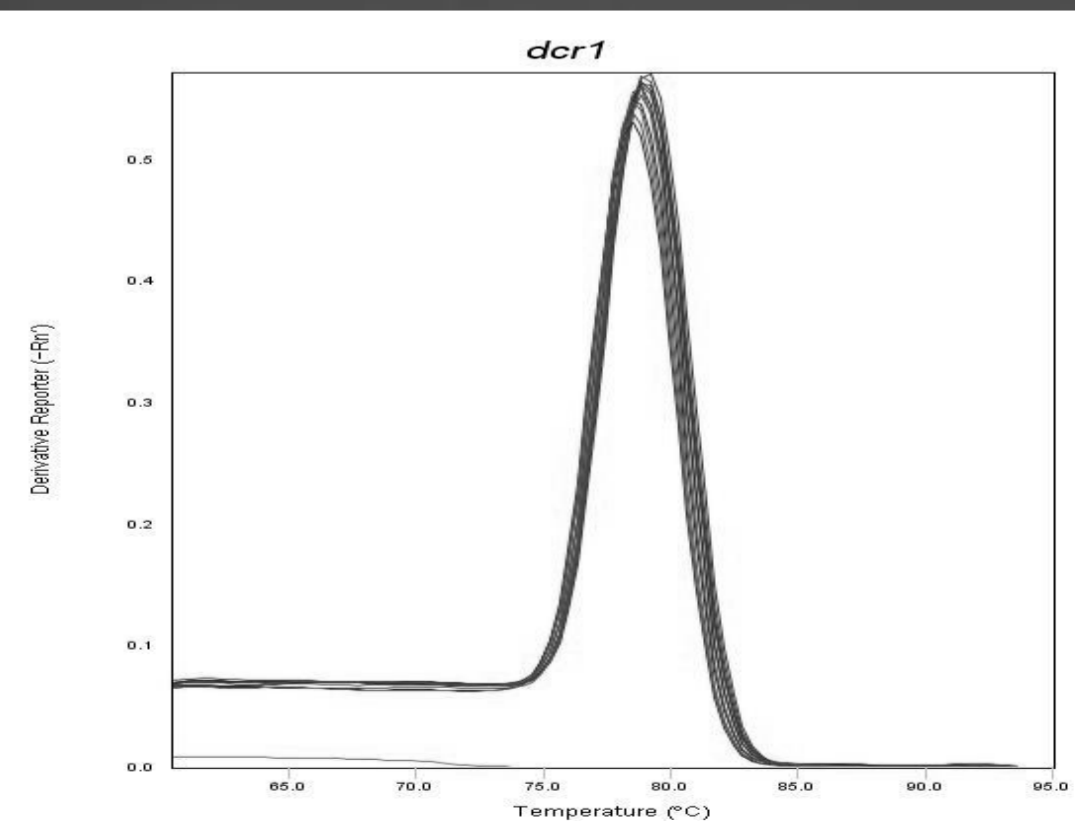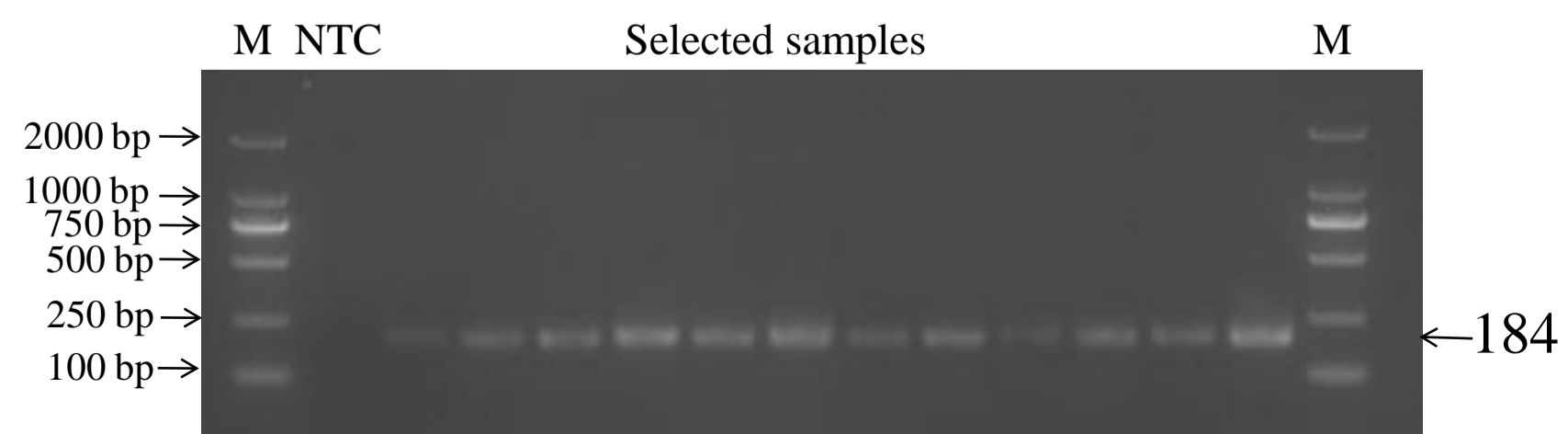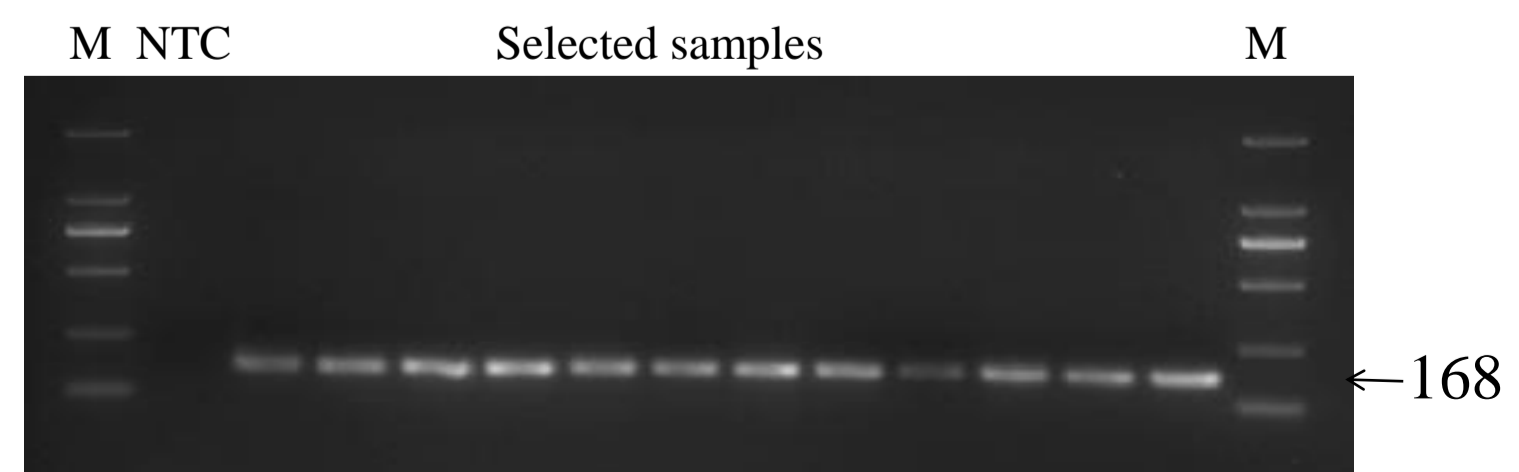

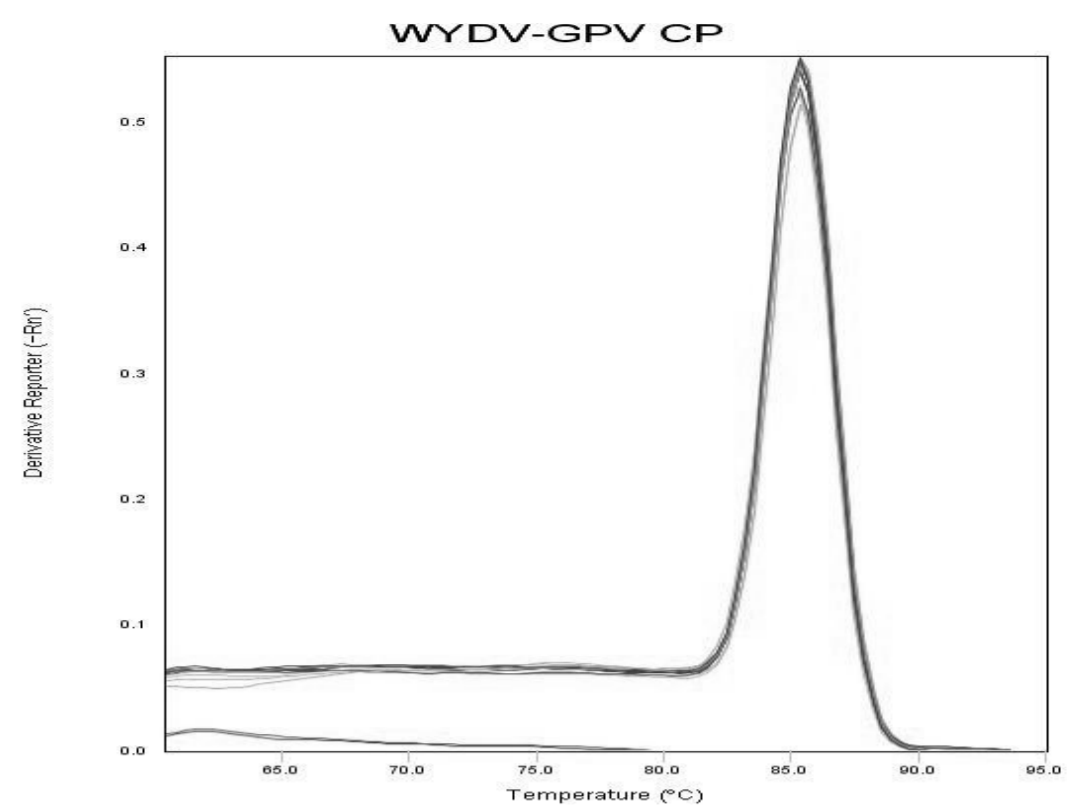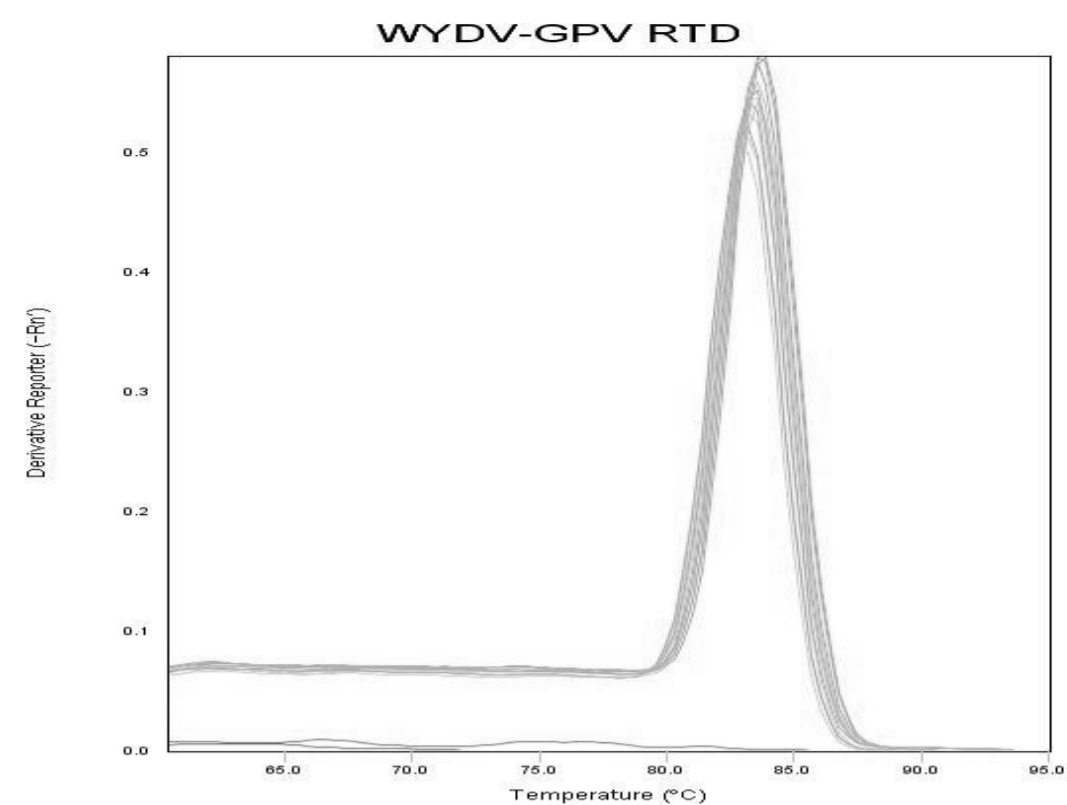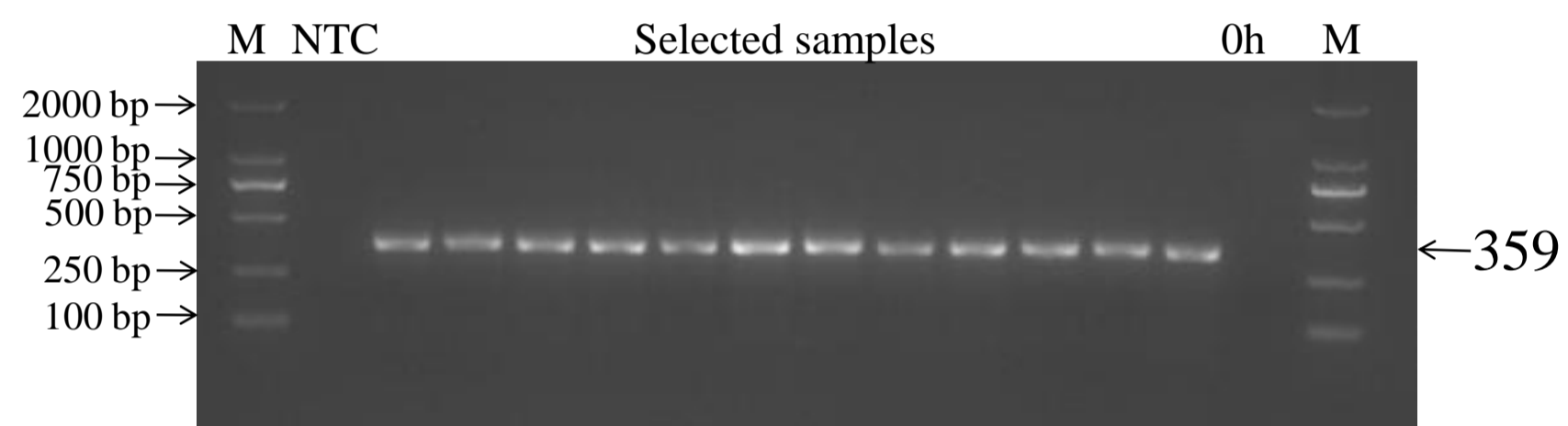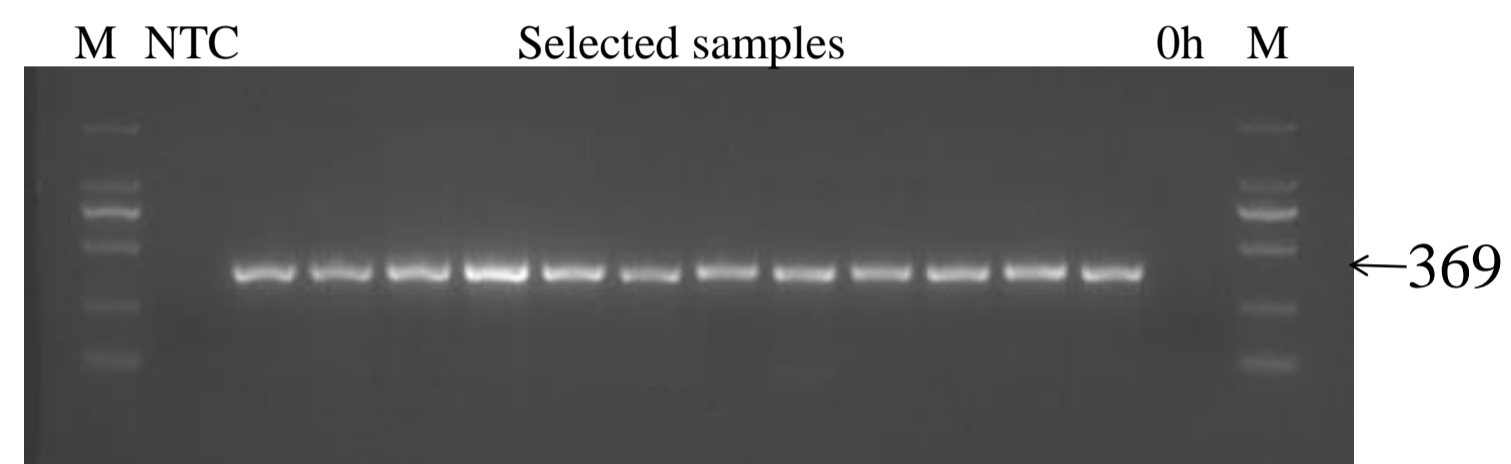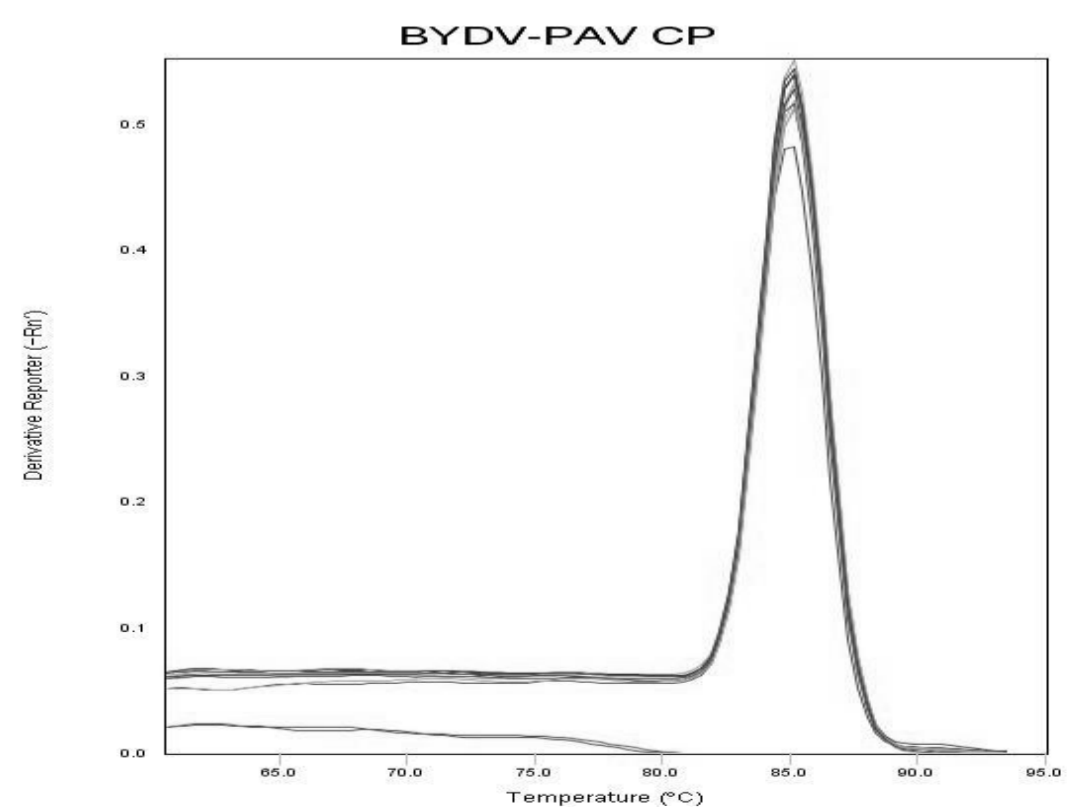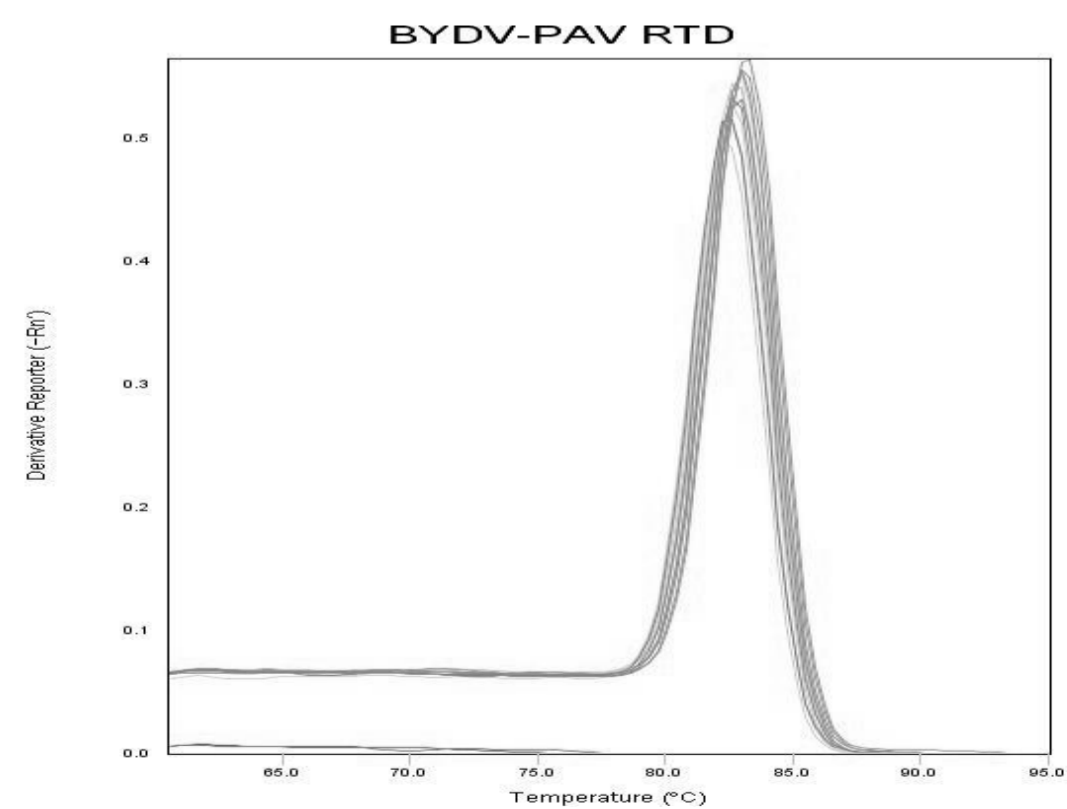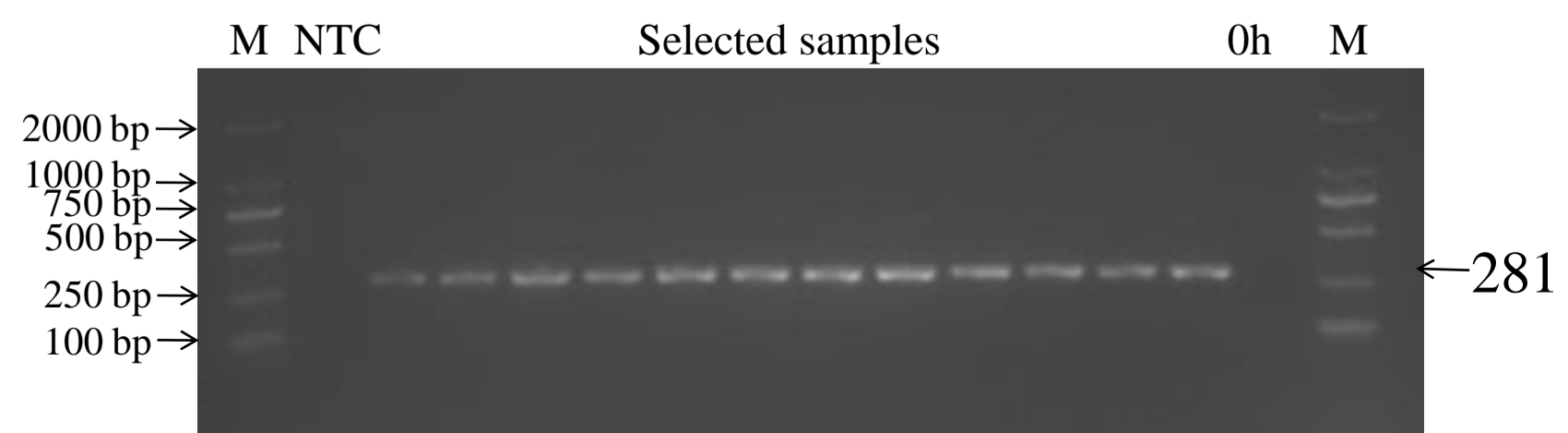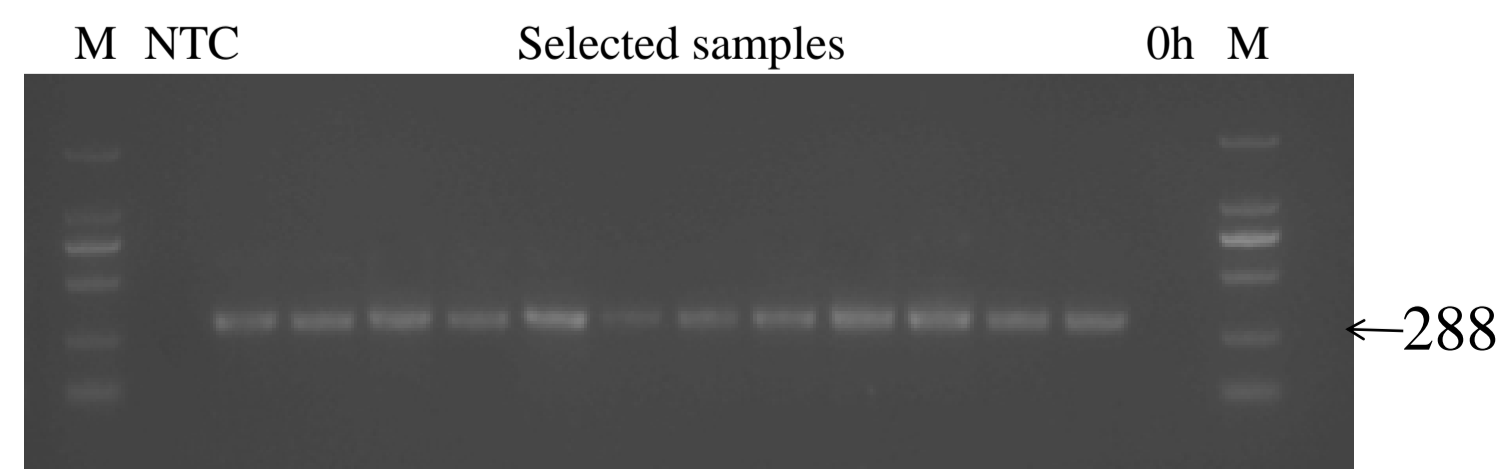

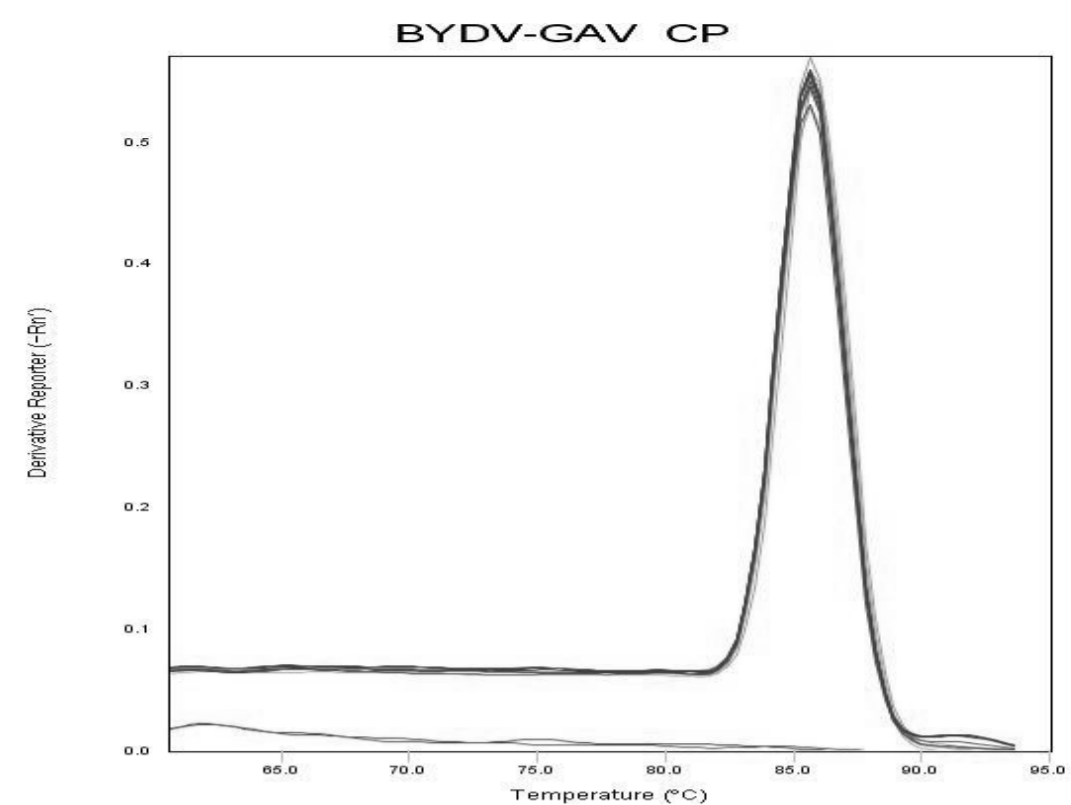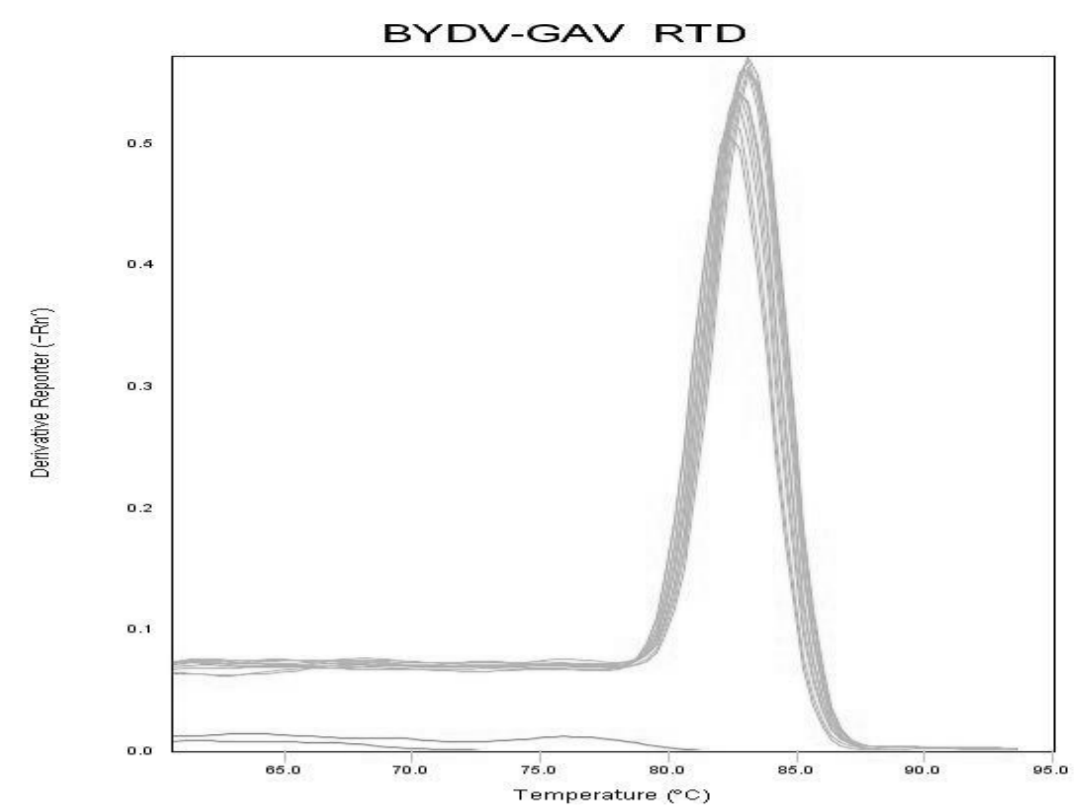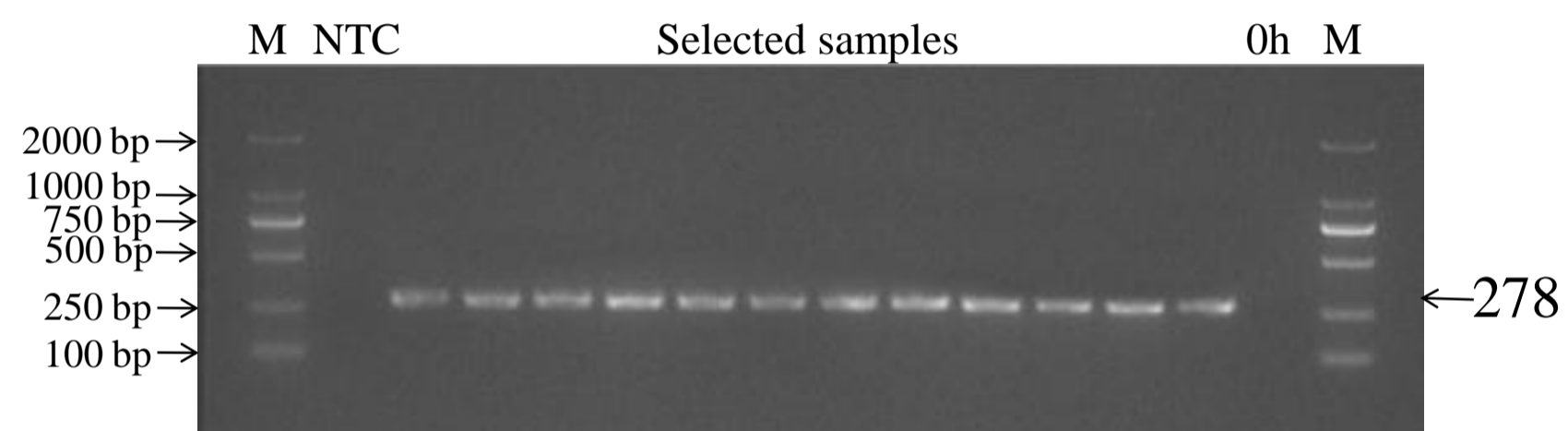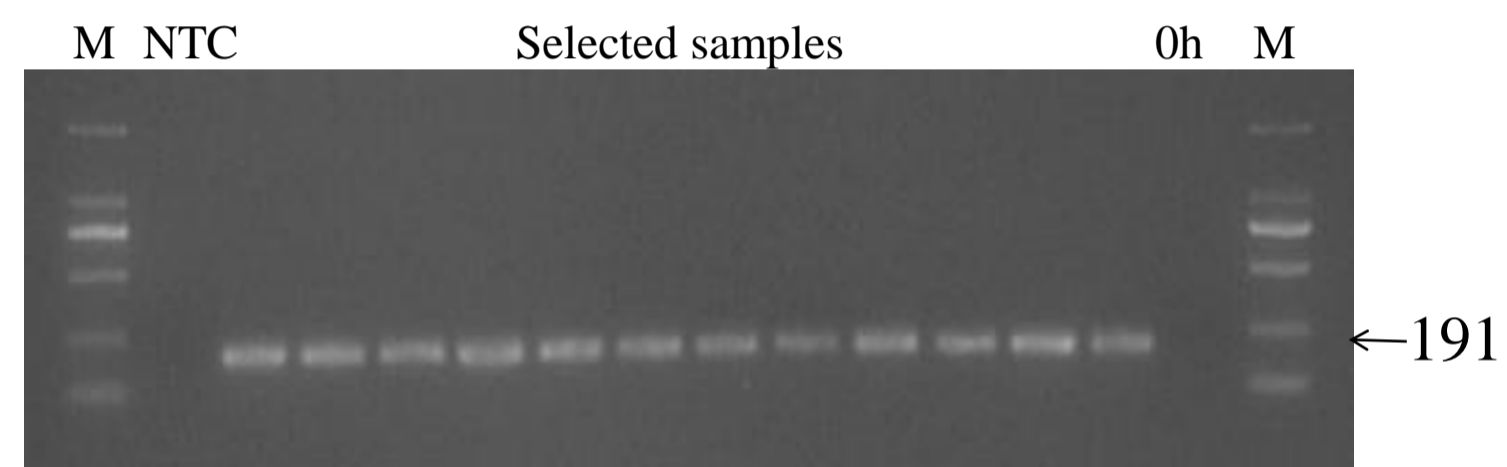

Supplement: Figure S3 — Melt curve and gel photo to check specificity and the size of RT-qPCR primer pair of each gene. NTC: no-template control; 0 h: 0 h feeding duration; selected samples: 12 randomly selected samples; M: DL2000. (PDF) [file pone.0097038.s003.pdf]
