# Supplementary material for: Sequencing and Validation of Reference Genes to Analyze Endogenous Gene Expression and Quantify Yellow Dwarf Viruses Using RT-qPCR in Viruliferous Rhopalosiphum padi
Source: PLoS One. 2014 May 8;9(5):e97038. doi: 10.1371/journal.pone.0097038 (PMC4014588; doi:10.1371/journal.pone.0097038)
Supplement: Figure S4 — Relative standard curves to determine amplification efficiencies of candidate reference genes and endogenous genes of the aphid Rhopalosiphum padi (diluted cDNA from virus-free winged and wingless adult aphids) and CP and RTD genes of YDVs (diluted cDNA from YDV-viruliferous winged and wingless aphids after a 12-h virus-feeding. (PDF) [file pone.0097038.s004.pdf]

Relative standard curves to determine amplification efficiency for each gene

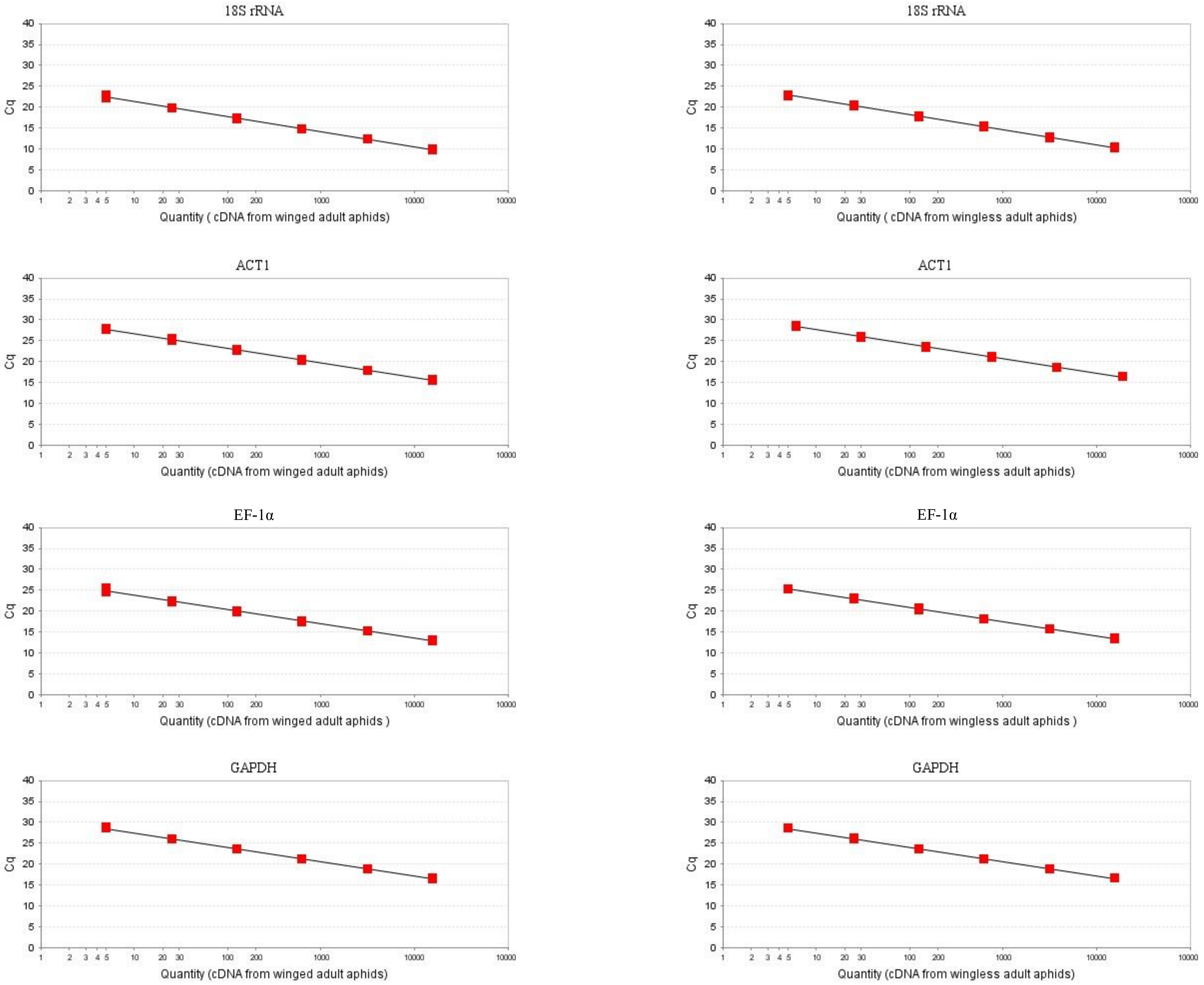

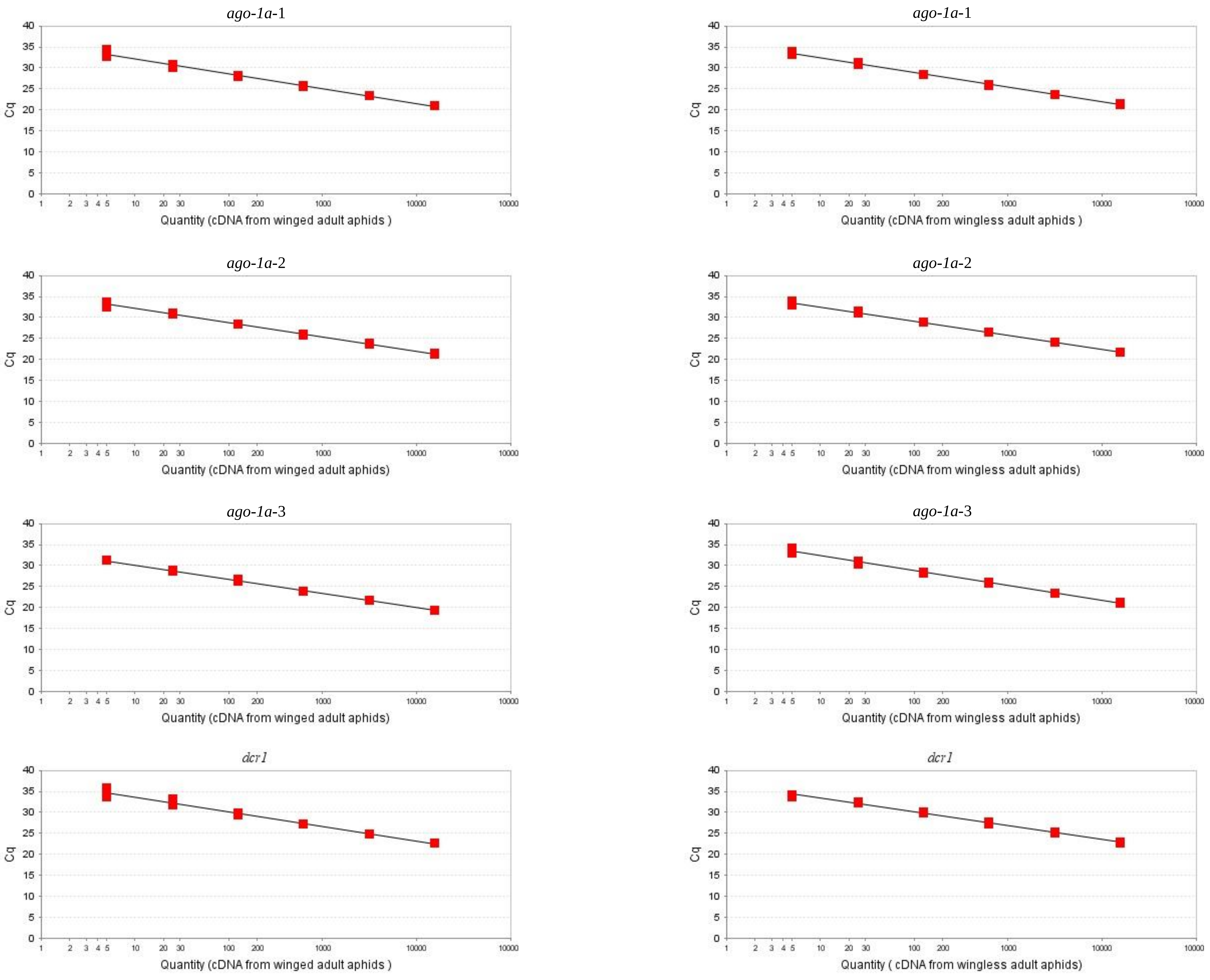

ago-1a-1
Cq
Quantity (cDNA from winged adult aphids)
ago-1a-1
Cq
Quantity (cDNA from wingless adult aphids)
ago-1a-2
Cq
Quantity (cDNA from winged adult aphids)
ago-1a-2
Cq
Quantity (cDNA from wingless adult aphids)
ago-1a-3
Cq
Quantity (cDNA from winged adult aphids)
ago-1a-3
Cq
Quantity (cDNA from wingless adult aphids)
dcr1
Cq
Quantity (cDNA from winged adult aphids)
dcr1
Cq
Quantity (cDNA from wingless adult aphids)

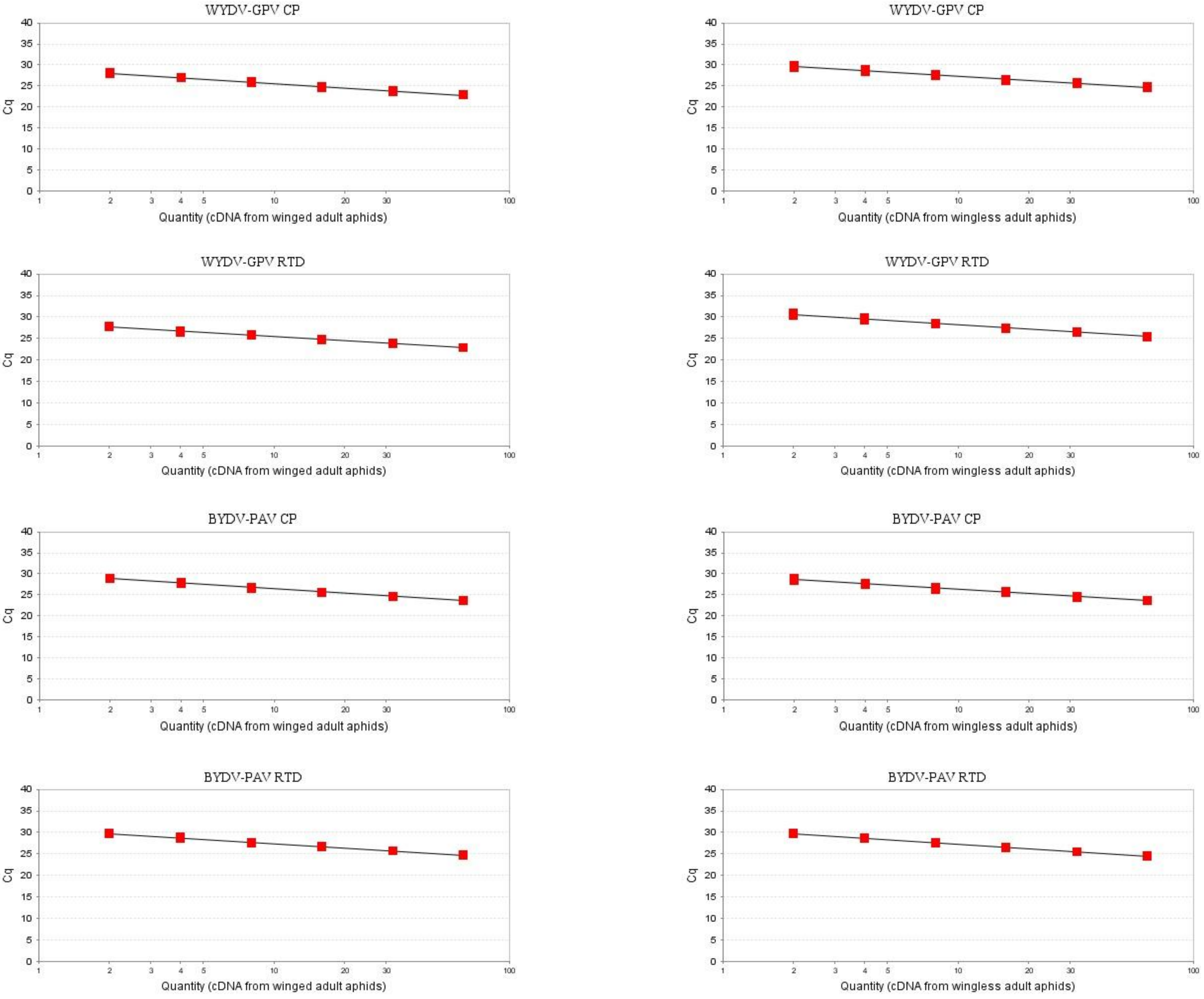
WYDV-GPV CP
Cq
Quantity (cDNA from winged adult aphids)
WYDV-GPV CP
Cq
Quantity (cDNA from wingless adult aphids)
WYDV-GPV RTD
Cq
Quantity (cDNA from winged adult aphids)
WYDV-GPV RTD
Cq
Quantity (cDNA from wingless adult aphids)
BYDV-PAV CP
Cq
Quantity (cDNA from winged adult aphids)
BYDV-PAV CP
Cq
Quantity (cDNA from wingless adult aphids)
BYDV-PAV RTD
Cq
Quantity (cDNA from winged adult aphids)
BYDV-PAV RTD
Cq
Quantity (cDNA from wingless adult aphids)

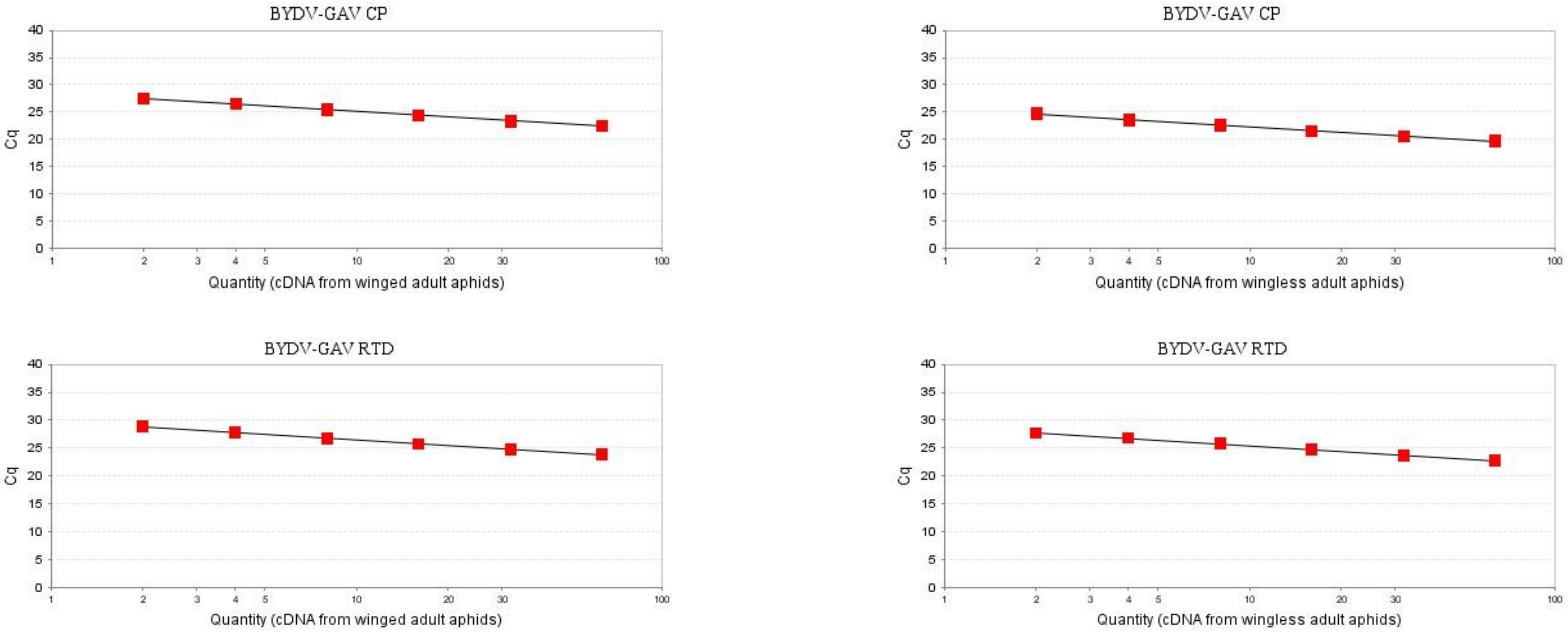
BYDV-GAV CP
Cq
Quantity (cDNA from winged adult aphids)
BYDV-GAV CP
Cq
Quantity (cDNA from wingless adult aphids)
BYDV-GAV RTD
Cq
Quantity (cDNA from winged adult aphids)
BYDV-GAV RTD
Cq
Quantity (cDNA from wingless adult aphids)

Description of relative standard curves

Gene	Slope (winged/wingless)	Intercept (winged/wingless)	R^2 (winged/wingless)	Efficiency (%) (winged/wingless)	Dilution range
18S rRNA	-3.573/-3.558	24.893/25.331	0.999/0.999	90.506/91.014	5-fold, 5^{-1}~5^{-6}
ACT1	-3.484/-3.446	30.22/31.119	0.999/0.999	93.665/95.067	5-fold, 5^{-1}~5^{-6}
EF-1α	-3.424/-3.392	27.284/24.679	0.998/0.999	95.902/97.157	5-fold, 5^{-1}~5^{-6}
GAPDH	-3.453/-3.416	30.962/30.936	0.999/1.000	94.814/96.216	5-fold, 5^{-1}~5^{-6}
ago-1a-1	-3.533/-3.541	35.733/36.038	0.991/0.997	91.891/91.594	5-fold, 5^{-1}~5^{-6}
ago-1a-2	-3.361/-3.345	35.494/35.865	0.996/0.997	98.391/99.05	5-fold, 5^{-1}~5^{-6}
ago-1a-3	-3.389/-3.537	33.556/35.831	0.999/0.994	97.263/91.749	5-fold, 5^{-1}~5^{-6}
dcr1	-3.478/-3.282	37.124/36.701	0.986/0.995	93.875/101.698	5-fold, 5^{-1}~5^{-6}
WYDV-GPV CP	-3.454/-3.278	29.059/30.621	0.996/0.992	94.75/101.875	2-fold, 2^{-1}~2^{-6}
WYDV-GPV RTD	-3.255/-3.416	28.757/31.599	0.994/0.989	102.875/96.203	2-fold, 2^{-1}~2^{-6}
BYDV-PAV CP	-3.484/-3.368	29.871/29.675	0.996/0.994	93.65/98.105	2-fold, 2^{-1}~2^{-6}
BYDV-PAV RTD	-3.315/-3.513	30.722/30.837	0.995/0.998	100.303/92.586	2-fold, 2^{-1}~2^{-6}
BYDV-GAV CP	-3.402/-3.308	28.555/25.615	0.999/0.997	96.752/100.563	2-fold, 2^{-1}~2^{-6}
BYDV-GAV RTD	-3.356/-3.319	29.842/28.765	0.998/0.996	98.585/100.127	2-fold, 2^{-1}~2^{-6}
