# Supplementary material for: Sequencing and Validation of Reference Genes to Analyze Endogenous Gene Expression and Quantify Yellow Dwarf Viruses Using RT-qPCR in Viruliferous Rhopalosiphum padi
Source: PLoS One. 2014 May 8;9(5):e97038. doi: 10.1371/journal.pone.0097038 (PMC4014588; doi:10.1371/journal.pone.0097038)
Supplement: Table S1 — Result of best Blast search hit of 18S rRNA, ACT1, EF-1α, and GAPDH. (PDF) [file pone.0097038.s005.pdf]

Table1 Result of best BLAST search hit

Query sequence/gene	Description	Max Score	Total Score	Query coverage	E value	Identity	Hit accession number
18S rRNA	Rhopalosiphum padi 18S ribosomal RNA gene, partial sequence	1031	1031	100%	0.0	100%	U27825.1
ACT1	Acyrthosiphon pisum actin (LOC100145822), mRNA	1837	1837	100%	0.0	96%	NM_001126200.2
EF-1α	Megoura crassicauda EF1a mRNA for elongation factor 1-alpha-like protein, partial cds, clone: Mcra-Tokyo-29	1285	1285	100%	0.0	94%	AB627794.1
GAPDH	PREDICTED: Acyrthosiphon pisum glyceraldehyde-3-phosphate dehydrogenase-like (LOC100169122), mRNA	1599	1599	100%	0.0	95%	XM_001943014.2
