# Supplementary material for: Sequencing and Validation of Reference Genes to Analyze Endogenous Gene Expression and Quantify Yellow Dwarf Viruses Using RT-qPCR in Viruliferous Rhopalosiphum padi
Source: PLoS One. 2014 May 8;9(5):e97038. doi: 10.1371/journal.pone.0097038 (PMC4014588; doi:10.1371/journal.pone.0097038)
Supplement: Table S2 — BestKeeper analysis of virus titre in YDV-viruliferous winged adults of Rhopalosiphum padi after different virus-feeding durations. n = 24: total number of samples used for analysis; Geo mean: geometric mean; Ar Mean: arithmetic mean; Min: minimun value of Cq; Max: maximum value of Cq; SD: standard deviation; CV: coefficient of variance. (PDF) [file pone.0097038.s006.pdf]

Table 2 BestKeeper analysis for the virus titre in YDVs-viruliferous winged adult of *Rhopalosiphum padi*

	**GPV-CP**	**GPV-RTD**	**PAV-CP**	**PAV-RTD**	**GAV-CP**	**GAV-RTD**
n	24	24	24	24	24	24
**Geo Mean [Cq]**	**25.36**	**25.92**	**25.07**	**25.72**	**25.18**	**25.44**
**Ar Mean [Cq]**	**25.39**	**25.96**	**25.12**	**25.77**	**25.19**	**25.45**
**Min [Cq]**	23.77	24.18	23.24	23.90	23.91	24.33
**Max [Cq]**	27.99	28.56	30.22	31.20	27.17	27.27
**SD [± Cq]**	**1.14**	**1.16**	**1.18**	**1.25**	**0.59**	**0.62**
**CV [% Cq]**	**4.48**	**4.47**	**4.68**	**4.87**	**2.35**	**2.42**
