# Supplementary material for: Sequencing and Validation of Reference Genes to Analyze Endogenous Gene Expression and Quantify Yellow Dwarf Viruses Using RT-qPCR in Viruliferous Rhopalosiphum padi
Source: PLoS One. 2014 May 8;9(5):e97038. doi: 10.1371/journal.pone.0097038 (PMC4014588; doi:10.1371/journal.pone.0097038)
Supplement: Table S3 — BestKeeper analysis of virus titre in YDV-viruliferous wingless adults of Rhopalosiphum padi after different virus-feeding durations. n = 24: total number of samples used for analysis; Geo mean: geometric mean; Ar Mean: arithmetic mean; Min: minimun value of Cq; Max: maximum value of Cq; SD: standard deviation; CV: coefficient of variance. (PDF) [file pone.0097038.s007.pdf]

Table 3 BestKeeper analysis for the virus titre in YDVs-viruliferous wingless adult of *Rhopalosiphum padi*

	GPV-CP	GPV-RTD	PAV-CP	PAV-RTD	GAV-CP	GAV-RTD
n	24	24	24	24	24	24
**Geo Mean [Cq]**	**25.02**	**25.76**	**24.20**	**25.23**	**24.20**	**24.33**
**Ar Mean [Cq]**	**25.05**	**25.79**	**24.25**	**25.28**	**24.22**	**24.35**
**Min [Cq]**	23.34	24.02	22.22	23.14	22.67	22.51
**Max [Cq]**	28.30	28.97	28.26	29.20	26.68	26.29
**SD [± Cq]**	**1.02**	**1.08**	**1.32**	**1.36**	**0.87**	**0.85**
**CV [% Cq]**	**4.08**	**4.19**	**5.46**	**5.36**	**3.61**	**3.50**
